# Pemt Inhibition‐Mediated Vdac1 Oligomerization Regulates Mitochondrial Dysfunction, Apoptosis, and Inflammation in High‐Fat Diet‐Derived Liver Injury

**DOI:** 10.1002/advs.76060

**Published:** 2026-07-02

**Authors:** Xianyong Bu, Xiufei Cao, Xin Yao, Baolin Li, Wencong Lai, Zengqi Zhao, Tingting Hao, Zhiwei Chen, Jianlong Du, Yueru Li, Kangsen Mai, Qinghui Ai

**Affiliations:** ^1^ Key Laboratory of Aquaculture Nutrition and Feed (Ministry of Agriculture and Rural Affair) Key Laboratory of Mariculture (Ministry of Education) Ocean University of China Qingdao P. R. China

**Keywords:** apoptosis, high‐fat diet, mitochondrial dysfunction, Nlrp3 inflammasome, Pemt, reactive oxygen species

## Abstract

High‐fat diet (HFD)‐induced hepatic injury represents a pathology observed across vertebrates, yet the underlying mechanisms remain incompletely understood. Herein, the present study identifies phosphatidylethanolamine methyltransferase (Pemt), a key enzyme in phosphatidylcholine synthesis, as a critical regulator of mitochondrial homeostasis in HFD‐driven liver injury. Our findings demonstrate that knockdown or knockout of *pemt* in hepatocytes from large yellow croaker (*Larimichthys crocea*) and in zebrafish (*Danio rerio*) liver markedly induces apoptosis, NOD‐like receptor protein 3 (Nlrp3) inflammasome activation, and mitochondrial dysfunction. Conversely, PEMT overexpression in large yellow croaker hepatocytes effectively attenuates palmitic acid‐induced apoptosis, Nlrp3 inflammasome activation, and mitochondrial dysfunction. Crucially, the present study reveals a direct protein–protein interaction between Pemt and voltage‐dependent anion channel 1 (Vdac1). Notably, VDAC1 overexpression significantly induces reactive oxygen species (ROS)‐dependent apoptosis and Nlrp3 inflammasome activation. Mechanistically, *pemt* deficiency enhances Vdac1 oligomerization, which in turn triggers apoptosis and Nlrp3 inflammasome activation in hepatocytes. Collectively, our results establish that HFD‐induced downregulation of Pemt promotes mitochondrial dysfunction and Vdac1 oligomerization, thereby exacerbating ROS‐dependent apoptosis and Nlrp3 inflammasome activation, ultimately leading to liver injury. Our findings establish the Pemt‐Vdac1 regulatory axis as a fundamental protective mechanism against overnutrition‐induced liver injury in vertebrates.

## Abstract 2

Pemt inhibition enhances Vdac1 oligomerization to drive mitochondrial dysfunction, apoptosis, and inflammation in high‐fat diet‐induced liver injury. The Pemt‐Vdac1 axis acts as a critical regulator in overnutrition‐induced hepatic damage in fish.

## 1 introduction

High‐fat diet (HFD)‐induced hepatic injury constitutes an evolutionarily conserved pathology across vertebrates, characterized by a progressive trajectory from lipid accumulation to inflammatory tissue damage. In humans, metabolic dysfunction‐associated steatohepatitis (MASH), strongly associated with HFD and metabolic dysregulation, has emerged as the leading cause of liver‐related mortality worldwide [1]. Epidemiological data reveal that MASH affects over 25% of the global adult population, with progression to hepatic fibrosis, cirrhosis, and hepatocellular carcinoma posing a major public health burden [2, 3]. In murine models, HFD feeding (mimicking a Western‐type diet) consistently induces steatohepatitis and liver injury [4, 5, 6]. Notably, teleost fish, as evolutionarily lower vertebrates, exposed to HFD, exhibit analogous pathological features, including hepatic steatosis, elevated activities of serum alanine aminotransferase (ALT) and aspartate aminotransferase (AST), as well as upregulated expression of hepatic pro‐inflammatory markers [7, 8]. This cross‐species conservation highlights a fundamental vertebrate vulnerability to overnutrition. Recent advances demonstrate that targeting multiple pathways, such as oxidative stress, apoptosis, and NOD‐like receptor protein 3 (NLRP3) inflammasome activation, can ameliorate HFD‐induced steatohepatitis and liver injury [9, 10, 11]. Nevertheless, the underlying mechanisms of HFD‐driven hepatotoxicity in vertebrates are not well understood [12, 13], which has become a recent focus.

Phosphatidylcholine (PC), the predominant mitochondrial phospholipid, is partially biosynthesized through phosphatidylethanolamine methyltransferase (PEMT)‐mediated tri‐methylation of phosphatidylethanolamine [14, 15]. This enzymatic reaction occurs primarily in the endoplasmic reticulum and mitochondrial‐associated membranes (MAM) [16]. Notably, mitochondria lack endogenous PC biosynthesis capacity and thus depend on MAM‐mediated interorganelle transport to maintain lipid homeostasis [17]. Previous clinical evidence suggests that MASH patients exhibit significantly reduced gene expression of hepatic *PEMT* [18]. Meanwhile, severe symptoms of steatohepatitis and liver damage were found in HFD‐fed *Pemt* knockout mice in animal experiments [19]. These collective findings position PEMT as a promising therapeutic target for mitigating HFD‐induced liver injury. However, the underlying mechanism of PEMT involved in HFD‐induced liver injury still needs to be fully elucidated.

Mitochondrial‐targeted therapies have emerged as a promising avenue for treating multiple metabolic syndromes such as diabetes, heart disease, and MASH [20]. As the primary energy‐producing organelles, mitochondria play a central role in determining hepatocyte metabolism and cellular fate [21]. Under conditions of chronic lipid overload, mitochondria initially compensate by enhancing fatty acid β‐oxidation (FAO) to maintain metabolic homeostasis [22]. However, sustained metabolic stress leads to electron transport chain (ETC) overloading, resulting in reactive oxygen species (ROS) production and consequent oxidative damage, constituting a critical molecular driver of HFD‐induced liver injury [23, 24]. Increasing evidence suggests that mitochondrial dysfunction‐induced ROS production serves as a critical trigger for both NLRP3 inflammasome activation and apoptotic pathways [25, 26, 27]. The activation of the NLRP3 inflammasome promotes CASPASE1‐mediated cleavage of pro‐interleukin‐1β (IL‐1β) to its mature form, which amplifies pro‐inflammatory signaling cascades and exacerbates liver injury [28]. Meanwhile, ROS accumulation disrupts mitochondrial membrane integrity, facilitating cytochrome C release through mitochondrial permeability transition pore (mPTP) opening [29]. This activates the intrinsic apoptotic pathway through CASPASE9 activation, which subsequently cleaves effector CASPASE3/7 activation, driving DNA fragmentation and hepatocyte demise [30]. The activated CASPASE3 drives apoptotic progression by proteolyzing poly ADP‐ribose polymerase 1 (PARP1), and PARP1 cleavage irreversibly terminates DNA repair functions, committing cells to programmed death [31]. Furthermore, an elevated BAX/BCL2 ratio enhances CASPASE9 activation efficiency, establishing a positive feedback loop that intensifies apoptotic signaling [32]. Although several mitochondrial‐targeting interventions, including antioxidants [33], plant extracts [27], and pioglitazone [34], have shown therapeutic potential against overnutrition‐induced liver injury, the underlying mechanisms remain incompletely understood. Notably, maintaining mitochondrial membrane structural and functional homeostasis has been recognized as a crucial strategy for mitigating oxidative stress, inflammation, and apoptosis [35, 36, 37]. A previous study reported that HFD‐fed *Pemt* knockout mice exhibited decreased mitochondrial cardiolipin content, a signature phospholipid of mitochondrial membranes, and exhibited cold‐intolerant [15]. Deficiency of *Pemt* in mice has been reported to lead to significant downregulation of ETC proteins [19]. These findings highlight the important role of *pemt* in maintaining mitochondrial function. Meanwhile, the present study identified voltage‐dependent anion channel‐1 (Vdac1) as an interacting partner of Pemt. Recent studies have revealed that VDAC1, residing in the outer mitochondrial membrane, can act as a mitochondrial gatekeeper and play a central hub role in metabolism, apoptosis, and inflammation, especially NLRP3 inflammasome activation [38]. Experiments in mice have revealed that targeting the VDAC1 protein is an important potential therapeutic for alleviating the pathological symptoms of steatohepatitis [39]. However, whether VDAC1 cooperatively modulates mitochondrial function with PEMT, thereby influencing the progression and severity of liver injury, requires further investigation.

Among the vertebrates, fish are the most ancient, diverse, and widely distributed vertebrate group worldwide [40]. Despite approximately 400 million years of phylogenetic divergence, teleosts and mammals maintain striking conservation in their metabolic processes and immune response pathways [41, 42]. Our previous works have indicated that large yellow croaker (*Larimichthys crocea*), a vital teleost in China, is highly conserved in regulation in lipid metabolism and inflammation compared to mammals, and could be a good model for studying HFD‐induced adverse symptoms and their mechanisms [43, 44]. Zebrafish (*Danio rerio*) is also a powerful vertebrate animal model, because zebrafish offers exceptional experimental advantages including low cost of feeding, high spawning capacity, short developmental cycle, and maturity of gene editing technology in numerous disease models [45]. Therefore, the current study used large yellow croaker and *pemt* knockout (*pemt* ^−/−^) zebrafish as vertebrate models to explore the role and underlying mechanisms of Pemt in HFD‐induced hepatotoxicity. Importantly, we identified the oligomerized state of Vdac1 as a novel target for Pemt‐mediated HFD‐induced mitochondrial dysfunction, apoptosis, and inflammatory responses. These findings provide fundamental insights into vertebrate metabolic regulation and identify novel therapeutic targets for overnutrition‐related pathologies.

## 2 Results

### 2.1 HFD Induces Liver Injury and Inhibits Hepatic Pemt Expression in Large Yellow Croaker

**FIGURE 1:**
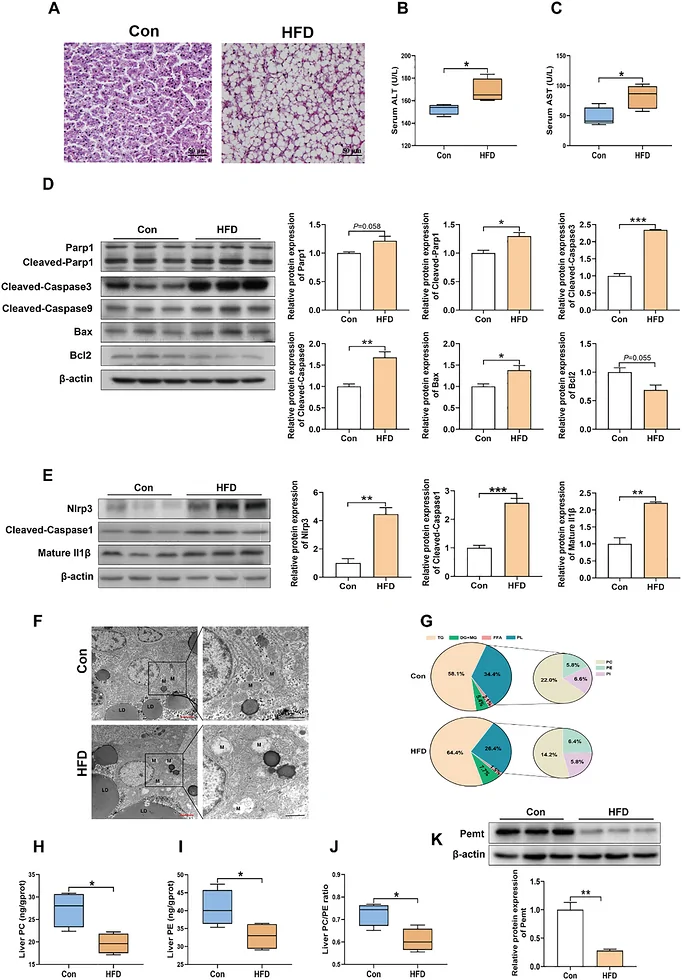
HFD diet induces liver injury and inhibits hepatic Pemt expression in large yellow croaker. (A) HE staining of liver sections in the Con and HFD groups (*n* = 4). Scale bars, 50 µm. (B) Serum alanine aminotransferase (ALT) and (C) aspartate aminotransferase (AST) activities in the Con and HFD groups (*n* = 6). (D) The protein expression of apoptosis‐related protein (Parp1, Cleaved‐Parp1, Cleaved‐Caspase3, Cleaved‐Caspase9, Bax, and Bcl2) (*n* = 3) and (E) Nlrp3 inflammasome activation‐related protein (Nlrp3, Cleaved‐Caspase1, and mature Il1β) in the liver of fish fed Con and HFD diets (*n* = 3). (F) Mitochondrial structure in the liver identified by TEM in fish fed Con and HFD diets. Red scale bars, 2 µm, black scale bars, 1 µm. M, mitochondria, LD, lipid droplet, N, nucleus. (G) Lipid class composition (TG, Diacylglycerol (DG), monoacylglycerol (MG), free fatty acids (FA), and phospholipids, including PC, PE, and phosphatidylinositol (PI)) in the liver identified by thin‐layer chromatography (TLC) in fish fed Con and HFD diets. (H) The PC, (I) PE, and (J) PC/PE ratio measured by enzyme‐linked immunosorbent assay kits in fish fed Con and HFD diets (*n* = 6). (K) Pemt protein expression in the liver of fish fed Con and HFD diets (*n* = 3). The results are presented as the mean ± SEM and analyzed by independent *t*‐tests. ^*^ *p* < 0.05, ^**^ *p* < 0.01, and ^***^ *p* < 0.001.

To establish an HFD‐induced liver injury model, large yellow croakers were fed HFD for 10 weeks. HFD‐fed fish exhibited markedly enhanced final body weight and hepatosomatic index (Figure S1A,B). Fish fed an HFD exhibited obvious lipid deposition in the liver, as evidenced by hepatic triglyceride (TG) content analysis and HE staining of liver sections (Figure 1A and Figure S1C), though no significant difference in hepatic total cholesterol (TC) content was observed (Figure S1D). Serum AST and ALT activities were markedly increased in the HFD group (Figure 1B,C), which indicated that HFD induced liver injury of large yellow croaker. Given the established roles of NLRP3 inflammasome activation and apoptosis in HFD‐induced liver injury [46, 47], the effect of HFD on hepatic protein expression related to Nlrp3 inflammasome activation and apoptosis was evaluated in large yellow croaker. HFD‐fed fish showed significantly upregulated hepatic protein expression of Cleaved‐Parp1, Cleaved‐Caspase3, Cleaved‐Caspase9, Bax, Nlrp3, Cleaved‐Caspase1, mature Il1β (Figure 1D,E), hepatic Caspase1 activity (Figure S1E), and serum Il1β content (Figure S1F), along with significantly up‐regulated gene expression of proinflammatory cytokine including tumor necrosis factor α (*tnfα*), cyclooxygenase‐2 (*cox2*), and interleukin 6 (*il6*) (Figure S1G). Similar effects in terms of inflammation and apoptosis were observed in large yellow croaker hepatocytes treated with palmitic acid (PA) (Figure S2A–E).

Mitochondrial damage is an important factor in causing NLRP3 inflammasome activation and apoptosis [26, 27]. In the present study, hepatic transmission electron microscope (TEM) observation demonstrated that fish fed with control diets showed normal mitochondrial structure with regularly arranged mitochondrial inner membrane cristae, while fragmented mitochondria with disrupted cristae were found in fish fed HFD (Figure 1F). This correlated with oxidative stress, as evidenced by reduced total antioxidant capacity (T‐AOC), glutathione (GSH) content, and total superoxide dismutase (T‐SOD) activity, and increased malondialdehyde (MDA) content in the liver of fish fed HFD (Figure S3). These findings collectively suggest a correlation between lipid overload and mitochondrial damage in fish. The lipid class and phospholipid composition were also analyzed. Fish fed HFD had obviously increased relative hepatic TG content and decreased phospholipid content, including reduced PC and PE levels, thereby lowering the PC/PE ratio (Figure 1G–J). Here, the present results also found that the *pemt* mRNA and Pemt protein expression underwent a significant reduction under HFD conditions (Figure 1K and Figure S4A), suggesting its potential role in HFD‐induced mitochondrial damage and liver injury.

### 2.2 Pemt Mediates PA‐Triggered Apoptosis and Inflammation in Hepatocytes of Large Yellow Croaker

**FIGURE 2:**
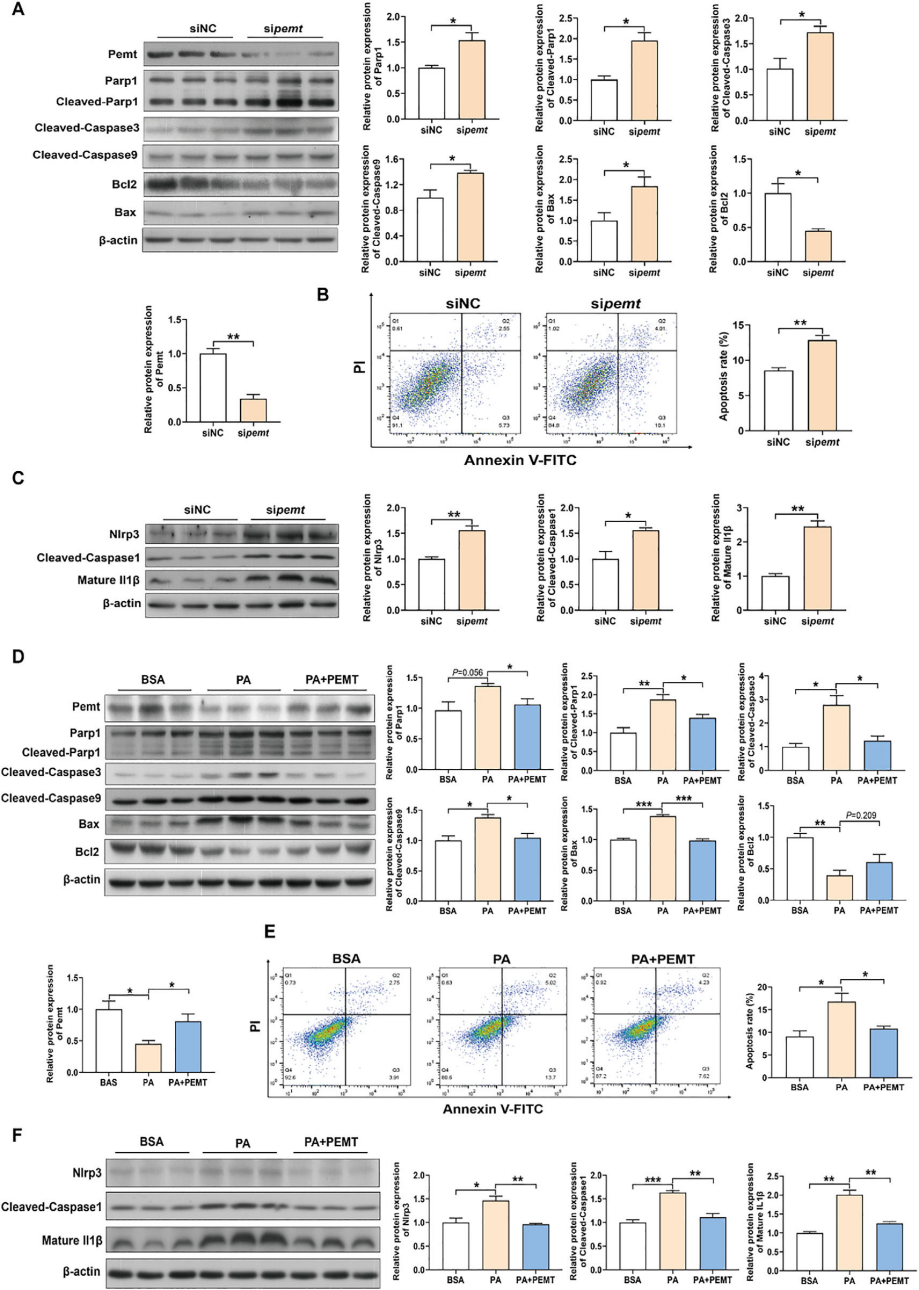
Pemt contributes to PA‐induced apoptosis and inflammation in large yellow croaker hepatocytes. (A) Protein expression of Pemt and apoptosis‐related protein (Parp1, Cleaved‐Parp1, Cleaved‐Caspase3, Cleaved‐Caspase9, Bax, and Bcl2) in the hepatocytes after transfected with si*pemt* (*n* = 3). (B) Apoptosis of the hepatocytes after transfected with si*pemt* was determined by flow cytometry (*n* = 3). (C) Nlrp3 inflammasome activation related protein (Nlrp3, cleaved‐Caspase1, and mature Il1β) expression in the hepatocytes after transfected with si*pemt* (*n* = 3). (D) Protein expression of Pemt and apoptosis‐related proteins (Parp1, Cleaved‐Parp1, Cleaved‐Caspase3, Cleaved‐Caspase9, Bax and Bcl2) in the hepatocytes after transfected with pcDNA3.1 and pcDNA3.1‐PEMT plasmid for 36 h, with or without subsequent 12 h incubation with PA (*n* = 3). (E) Apoptosis of the hepatocytes after transfected with pcDNA3.1 and pcDNA3.1‐PEMT plasmids for 36 h, with or without subsequent 12 h incubation with PA determined by flow cytometry (*n* = 3). (F) Nlrp3 inflammasome activation‐related protein (Nlrp3, Cleaved‐Caspase1, and mature Il1β) expression in the hepatocytes after transfected with pcDNA3.1 and pcDNA3.1‐PEMT plasmid for 36 h, with or without subsequent 12 h incubation with PA (*n* = 3). The results are presented as the mean ± SEM and analyzed by independent *t*‐tests. ^*^ *p* < 0.05, ^**^ *p* < 0.01, and ^***^ *p* < 0.001.

To investigate the effects of Pemt on apoptosis and inflammatory response, important markers of liver injury, *pemt* was successfully knocked down in the hepatocytes of large yellow croaker after being transfected with small interfering RNA (si*pemt*). The lowest *pemt* gene and Pemt protein expression levels were observed in the hepatocyte transfected with si*pemt*#3 (Figure S4B,C), thus si*pemt*#3 was used in subsequent experiments. As expected, *pemt* knockdown markedly upregulated the protein expression of apoptosis‐related proteins, including Parp1, Cleaved‐Parp1, Cleaved‐Caspase3, Cleaved‐Caspase9, and Bax, and decreased the protein expression of anti‐apoptotic protein Bcl2 (Figure 2A). Meanwhile, flow cytometry assay showed a visible increased apoptosis rate in the hepatocytes following *pemt* knockdown (Figure 2B). Also, hepatocyte protein expression of Nlrp3, Cleaved‐Caspase1, and mature‐Il1β, hepatocyte Caspase1 activity, Il1β content in the culture medium, and hepatocyte mRNA expression of *tnfa*, *cox2*, and *il8* were significantly increased when the hepatocytes were treated with si*pemt* (Figure 2C and Figure S5A–C). Therefore, Pemt suppression could exacerbate apoptosis and inflammatory response in large yellow croaker hepatocytes. Further, hepatocytes were transfected with pcDNA3.1 or pcDNA3.1‐PEMT plasmids by electroporation followed by PA treatment. Overexpression of PEMT significantly reversed PA‐induced reduction of Pemt protein expression. The apoptosis rate and protein expression of apoptosis‐related proteins, including Parp1, Cleaved‐Parp1, Cleaved‐Caspase3, Cleaved‐Caspase9, and Bax, were significantly reduced after PEMT was overexpressed under PA treatment (Figure 2D–E). Consistent with this, overexpression of PEMT also markedly reversed PA‐induced increases in hepatocyte protein expression of Nlrp3, Cleaved‐Caspase1, and mature Il1β, hepatocyte Caspase1 activity, Il1β content in the culture medium, and mRNA expression of *tnfa*, *cox2*, and *il6* in the hepatocytes of large yellow croaker (Figure 2F; Figure S5D–F). Together, these data indicate that PA‐mediated downregulation of Pemt promotes apoptosis and inflammation in hepatocytes of large yellow croaker.

### 2.3 *pemt* Deficiency Induces Apoptosis and Inflammatory Responses in the Liver of Zebrafish

**FIGURE 3:**
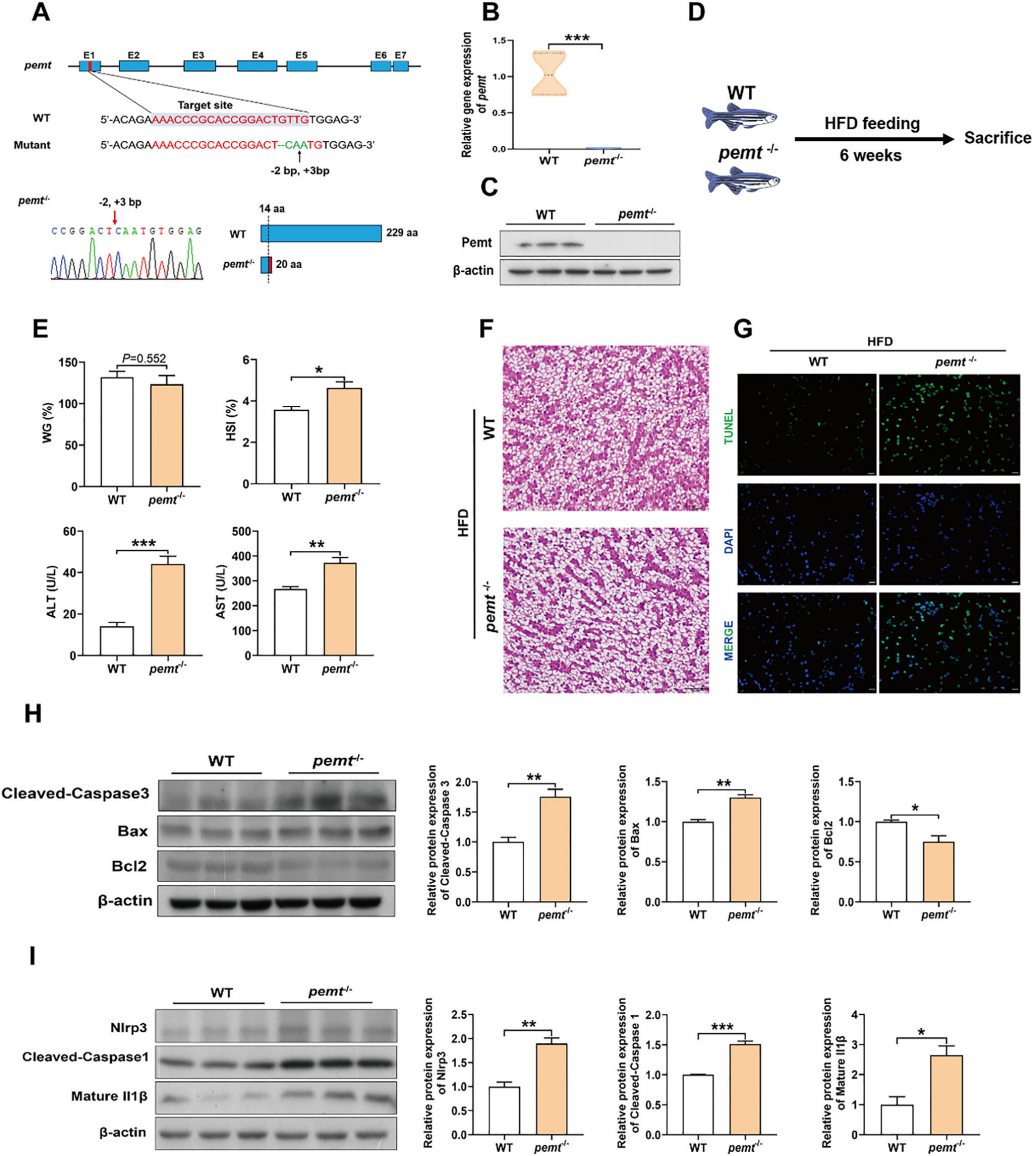
*pemt* knockout promotes apoptosis and inflammatory responses in the liver of zebrafish. (A) Zebrafish *pemt* target site, the deduced peptide length of WT and *pemt* ^−/−^, and the DNA sequencing results of the mutant. E, exon. (B) Gene expression of *pemt* in the liver of WT and *pemt* ^−/−^ zebrafish (120 dpf) (*n* = 6). (C) Protein expression of Pemt in the liver of WT and *pemt* ^−/−^ zebrafish (120 dpf) (*n* = 3). (D) Experiment flowchart of *pemt* knockout induced liver injury of zebrafish. (E) The WG, HSI, and serum ALT and AST activities of WT and *pemt* ^−/−^ zebrafish (*n* = 6). (F) HE staining of liver sections in WT and *pemt* ^−/−^ zebrafish. Scale bars, 50 µm. (G) Apoptosis rate in the liver of WT and *pemt* ^−/−^ zebrafish determined by TUNEL staining. White scale bars, 10 µm. (H) Protein expression of apoptosis‐related protein (Cleaved‐Caspase3, Bax, and Bcl2) (*n* = 3) and (I) Nlrp3 inflammasome activation‐related protein (Nlrp3, Cleaved‐Caspase1, and mature Il1β) in the liver of WT and *pemt* ^−/−^ zebrafish (*n* = 3). The results are presented as the mean ± SEM and analyzed by independent *t*‐tests. ^*^ *p* < 0.05, ^**^ *p* < 0.01, and ^***^ *p* < 0.001.

To further confirm the effects of *pemt* deficiency on hepatic injury, apoptosis, and inflammatory responses in fish, the *pemt* knockout zebrafish model was established using CRISPR/Cas9 technology. The *pemt* ^−/−^ zebrafish was generated by adding 3 bp and deleting 2 bp from the first exon of *pemt*, resulting in a premature stop codon that shortened the encoded protein to only 20 amino acids (Figure 3A). Successful model establishment was confirmed by significant reductions in both *pemt* mRNA levels and Pemt protein expression (Figure 3B,C). Next, WT and *pemt* ^−/−^ zebrafish were fed formulated diets for 6 weeks (Figure 3D). There was no significant change in weight gain between *pemt* ^−/−^ and WT zebrafish. However, the *pemt* ^−/−^ zebrafish had significantly higher hepatosomatic index and activities of serum ALT and AST than those of WT zebrafish, demonstrating that *pemt* deficiency exacerbated liver injury in fish (Figure 3E). Furthermore, histological analysis coupled with TUNEL staining revealed obvious hepatocyte vacuolization and enhanced apoptosis in *pemt* ^−/−^ zebrafish (Figure 3F,G). Consistently, *pemt* ^−/−^ zebrafish demonstrated markedly upregulated protein expression of Cleaved‐Caspase3, Bax, Nlrp3, Cleaved‐Caspase1, and mature Il1β, concurrent with significantly decreased Bcl2 expression (Figure 3H,I). The hepatic Caspase1 activity and serum Il1β content of *pemt* ^−/−^ zebrafish were also increased compared with those of WT zebrafish (Figure S6A,B). Meanwhile, the gene expression of *tnfa*, *cox2*, and *il6* was significantly upregulated in the liver of *pemt* ^−/−^ zebrafish compared with that of WT zebrafish (Figure S6C). These findings collectively suggest that *pemt* deficiency exacerbates hepatic apoptosis and inflammatory responses in zebrafish, consistent with the results in large yellow croaker.

### 2.4 Pemt Maintains Mitochondrial Functional Homeostasis in Fish

**FIGURE 4:**
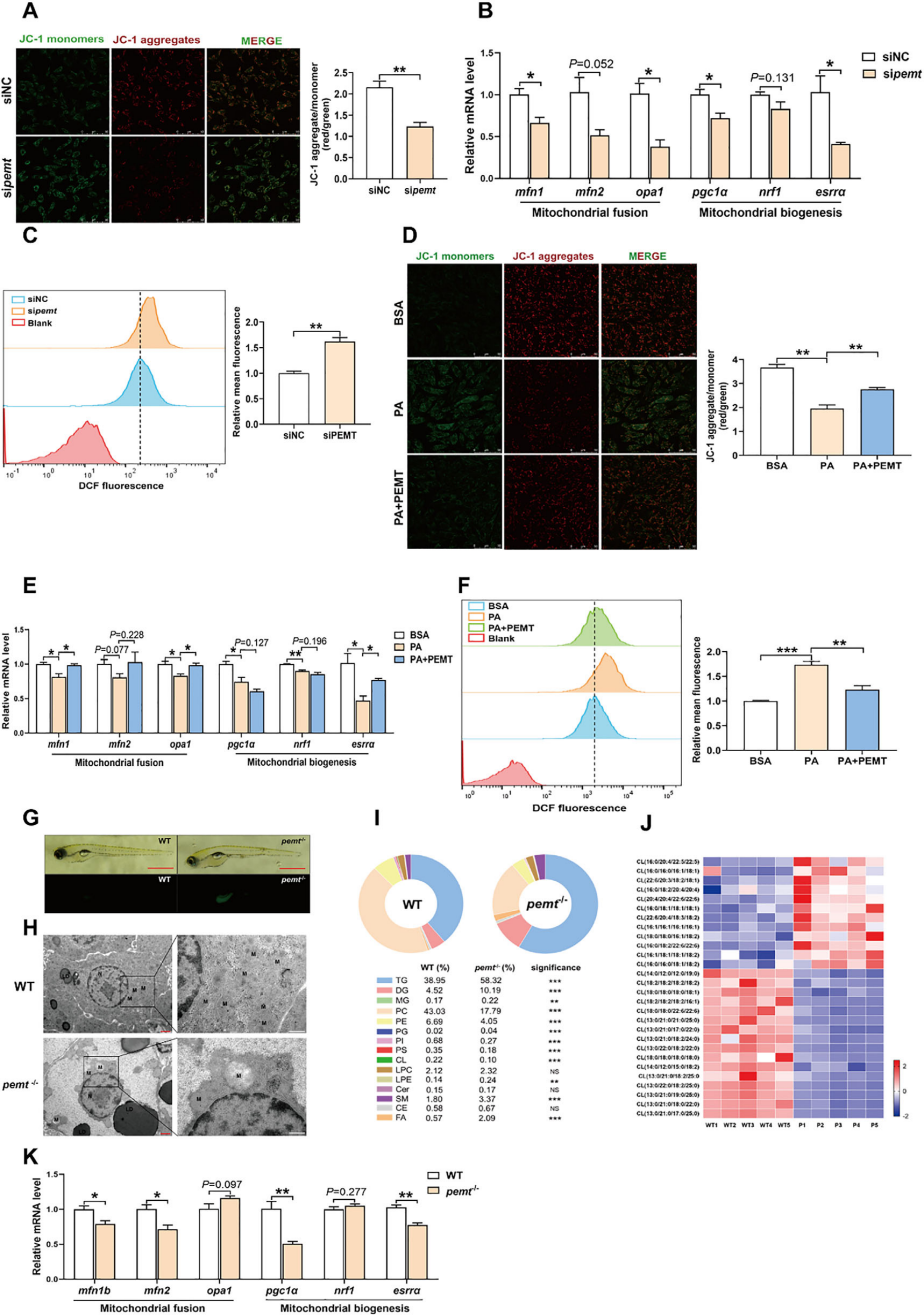
Pemt contributes to the maintenance of mitochondrial functional homeostasis in large yellow croaker and zebrafish. (A) The MMP in the hepatocytes of large yellow croaker after transfected with si*pemt* (*n* = 3). (B) Gene expression of mitochondrial fusion (*mfn1*, *mfn2*, and *opa1*) and mitochondrial biogenesis (*pgc1α*, *nrf1*, and *esrrα*) in the hepatocytes of large yellow croaker after transfected with si*pemt* (*n* = 3). (C) ROS levels of the large yellow croaker hepatocytes after transfected with si*pemt* determined by flow cytometry (*n* = 3). (D) The MMP in the hepatocytes of large yellow croaker after transfected with pcDNA3.1 and pcDNA3.1‐PEMT plasmid for 36 h, with or without subsequent 12 h incubation with PA (*n* = 3). (E) Gene expression of mitochondrial fusion (*mfn1*, *mfn2*, and *opa1*) and mitochondrial biogenesis (*pgc1α*, *nrf1*, and *esrrα*) in the hepatocytes of large yellow croaker after transfected with pcDNA3.1 and pcDNA3.1‐PEMT plasmid for 12 h, with or without subsequent 12 h incubation with PA (*n* = 3). (F) ROS levels of large yellow croaker hepatocytes after transfected with pcDNA3.1 and pcDNA3.1‐PEMT plasmids for 36 h, with or without subsequent 12 h incubation with PA determined by flow cytometry (*n* = 3). (G) DCFH‐DA staining of WT and *pemt* ^−/−^ zebrafish (15 dpf). Scale bars, 0.5 mm. (H) Mitochondrial structure in the liver identified by TEM in WT and *pemt* ^−/−^ zebrafish (120 dpf). Red scale bars, 1 µm, white scale bars, 0.5 µm. M, mitochondria, LD, lipid droplet, N, nucleus. (I) Relative levels of TG, DG and MG, PC, PE, phosphatidylglycerol (PG), PI, phosphatidylserine (PS), cardiolipin (CL), lysophosphatidylcholine (LPC), lysophosphatidylethanolamine (LPE), ceramide (Cer), sphingomyelin (SM), cholesterol ester (CE), and FA (*n* = 5) and (J) CL composition (The values in heatmap represent log2 fold changes. Red indicates a high level of cardiolipin, whereas blue indicates a low level.) in the liver of WT and *pemt* ^−/−^ zebrafish identified by lipidomic analysis (*n* = 5). (K) Gene expression of mitochondrial fusion (*mfn1b*, *mfn2*, and *opa1*) and mitochondrial biogenesis (*pgc1α*, *nrf1*, and *esrrα*) in the liver of WT and *pemt* ^−/−^ zebrafish (*n* = 6). The results are presented as the mean ± SEM and analyzed by independent *t*‐tests. ^*^ *p* < 0.05, ^**^ *p* < 0.01, and ^***^ *p* < 0.001.

Next, given that mitochondrial dysfunction is a key contributor to inflammatory responses and apoptosis [25, 27], the role of Pemt in maintaining mitochondrial functional homeostasis was analyzed. The mitochondrial membrane potential (MMP) is a key marker for assessing mitochondrial state. In the current study, a significant increase in green fluorescence was observed in large yellow croaker hepatocytes following *pemt* knockdown (Figure 4A), suggesting that mitochondria were damaged to a certain extent. Since mitochondrial fusion and biogenesis are essential for mitochondrial homeostasis [48], the mitochondrial biogenesis and fusion‐related gene expression after *pemt* knockdown was determined. The gene expression of mitofusin 1 (*mfn1*), optic atrophy 1 (*opa1*), peroxisome proliferator‐activated receptor γ coactivator 1α (*pgc1α*), and estrogen‐related receptor α (*esrrα*) was significantly downregulated in large yellow croaker hepatocytes subjected to *pemt* knockdown (Figure 4B). Meanwhile, flow cytometry assay showed a visible increased ROS production as indicated by elevated relative mean fluorescence in the hepatocytes following *pemt* knockdown (Figure 4C), suggesting that *pemt* knockdown exacerbated ROS production and mitochondrial dysfunction. In contrast, to investigate whether PEMT overexpression could ameliorate PA‐induced mitochondrial dysfunction, PEMT was overexpressed in large yellow croaker hepatocytes by transfection with the pcDNA3.1‐PEMT plasmid following PA treatment. The results in the present study showed that the MMP, gene expression of *mfn1*, *opa1*, and *esrrα*, and ROS production were obviously reversed by PEMT overexpression (Figure 4D–F). Overall, these results indicated that Pemt plays a crucial role in PA‐induced mitochondrial dysfunction in large yellow croaker hepatocytes.

To further confirm the influences of *pemt* deficiency on mitochondrial homeostasis in fish, the ROS production, mitochondrial structure, lipid composition, and mitochondrial fusion and biogenesis were determined in *pemt* ^−/−^ and WT zebrafish. The fluorescent probe 2’, 7’‐dichlorodihydrofluorescein diacetate (DCFH‐DA) staining showed that *pemt* ^−/−^ zebrafish exhibited higher ROS production than that in the WT zebrafish (Figure 4G). Furthermore, approximately complete and uniformly sized mitochondria were observed in WT zebrafish, while swollen and inner membrane‐damaged mitochondria were detected in *pemt* ^−/−^ zebrafish (Figure 4H). Notably, lipidomic analysis revealed that hepatic cardiolipin, a characteristic phospholipid of mitochondria [49], was significantly reduced in *pemt* ^−/−^ zebrafish, especially in cardiolipin containing four C18:2 acyl chains (Figure 4I,J). Consistent with this, the gene expression of *mfn1b*, *mfn2*, *pgc1α*, and *esrrα* was significantly downregulated in the liver of *pemt* ^−/−^ zebrafish compared with that in the WT zebrafish (Figure 4K). Overall, these findings confirm that *pemt* deficiency disrupts mitochondrial structure, leading to mitochondrial dysfunction and excessive ROS production.

### 2.5 Identification of Vdac1 as a Pemt‐Interacting Protein in Large Yellow Croaker

**FIGURE 5:**
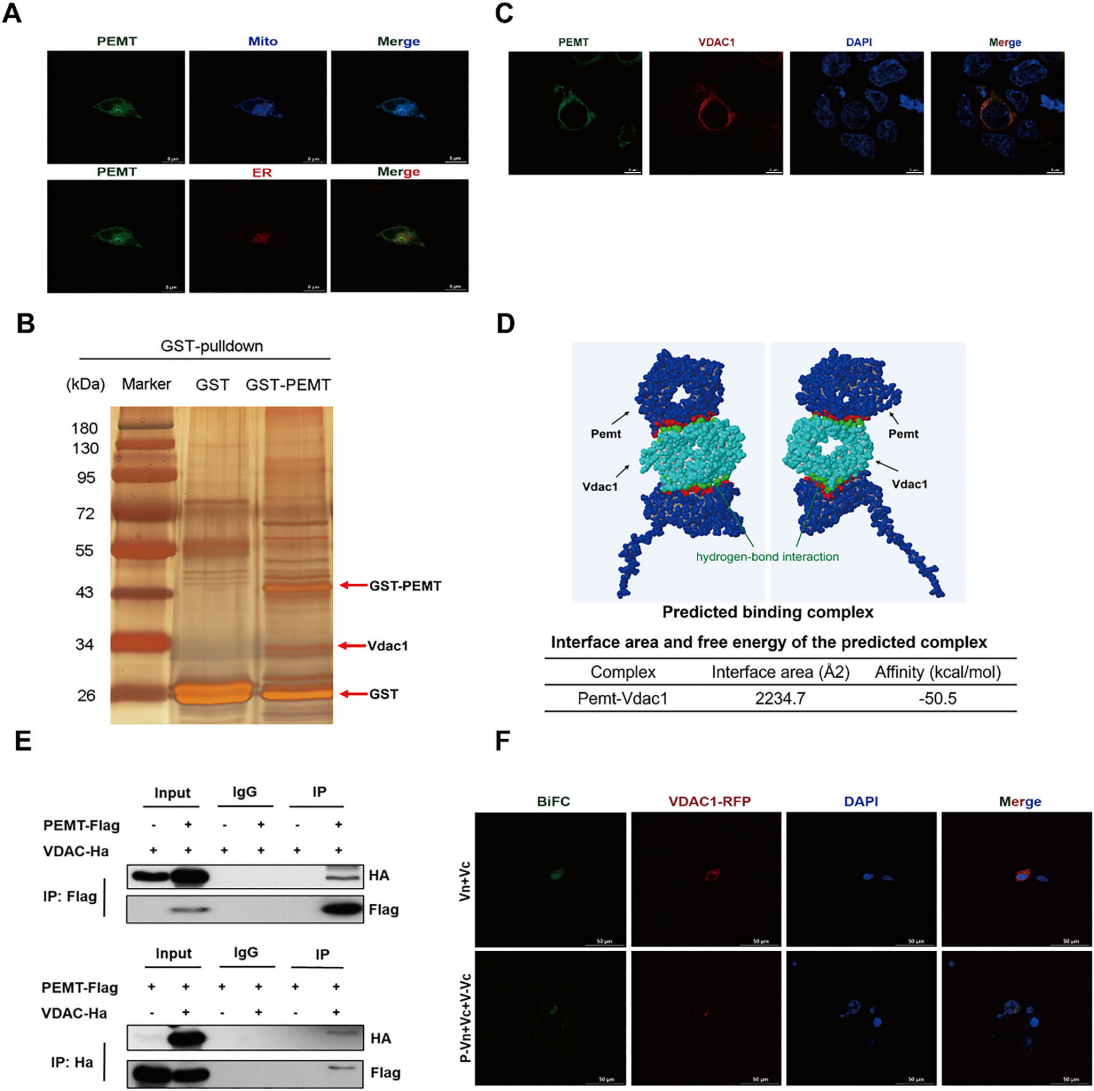
Identification of Vdac1 as a Pemt‐interacting protein in large yellow croaker. (A) The subcellular distribution of Pemt in the mitochondria and endoplasmic reticulum of large yellow croaker. Scale bars, 8 µm. (B) GST‐pulldown assay using large yellow croaker hepatocytes. (C) Colocalization of Pemt and Vdac1 of large yellow croaker in the HEK293T co‐transfected with pcDNA3.1‐PEMT‐GFP and pcDNA3.1‐VDAC1‐RFP plasmids for 24 h. Scale bars, 8 µm. (D) The 3D structures of large yellow croaker Pemt and Vdac1 were obtained from SWISS‐MODEL, and their binding complex was predicted using the PDBePISA docking tool. Atoms are color‐coded as follows: gray (hydrogen), green (carbon), red (oxygen), and blue (nitrogen). The interaction interface area and binding free energy of the predicted complex were shown in the table. A larger interface area facilitates protein‐protein binding, while a negative free energy value indicates stable complex formation. (E) The interaction between Pemt and Vdac1 determined by co‐immunoprecipitation analysis. (F) The interaction between Pemt and Vdac1 determined by a bimolecular fluorescence complementation assay. Vn, pBiFC‐VN173, Vc, pBiFC‐VC155, P‐Vn, pBiFC‐VN173‐PEMT, V‐Vc, pBiFC‐VC155‐VDAC1.

Subcellular localization results showed that Pemt was expressed in both mitochondria and endoplasmic reticulum, suggesting that Pemt was localized to the MAM (Figure 5A). Given the crucial role of mitochondrial outer membrane protein VDAC1 in oxidative stress, inflammatory responses, and apoptosis [50], whether Vdac1 could serve as a Pemt‐interacting protein was explored in the present study. Interestingly, the GST‐pulldown assay showed that Vdac1 could interact with Pemt (Figure 5B). The co‐localization analysis by confocal microscopy confirmed a significant overlap between Vdac1 and Pemt (Figure 5C). Subsequently, protein–protein docking predicted their 3D complex structure, revealing a stable interaction primarily through hydrogen bonds, as evidenced by low binding free energy (Figure 5D). Co‐immunoprecipitation and BIFC analyses further confirmed the Pemt‐Vdac1 interaction in large yellow croaker (Figure 5E,F), suggesting that Pemt may influence downstream pathological changes by interacting with Vdac1.

### 2.6 Vdac1‐ROS Pathway Mediates PA‐Induced Apoptosis and Inflammation in Hepatocytes of Large Yellow Croaker

**FIGURE 6:**
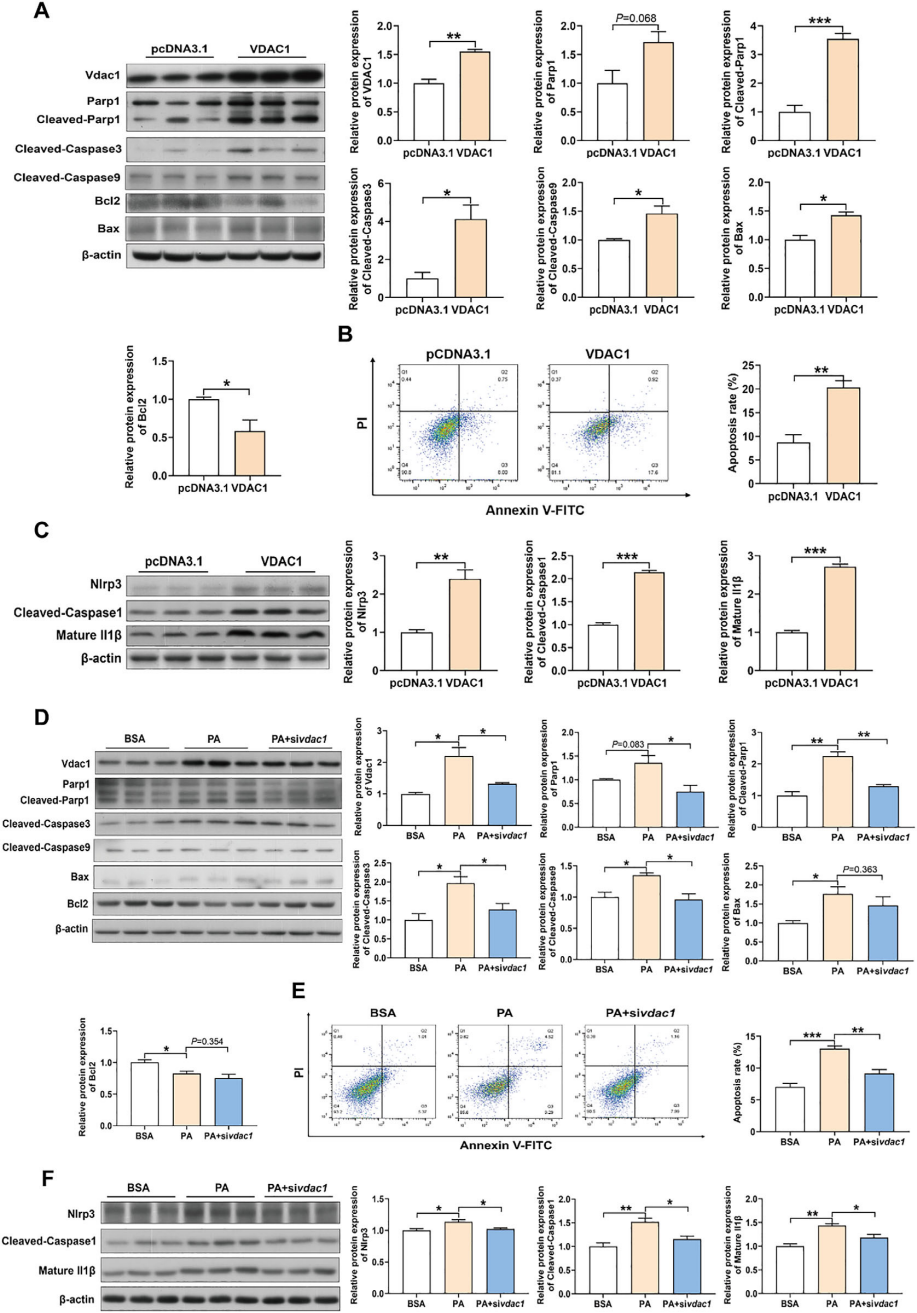
Vdac1 contributes to palmitic acid‐induced apoptosis and inflammation in hepatocytes of large yellow croaker. (A) Expression of Vdac1 and apoptosis related protein (Parp1, Cleaved‐Parp1, Cleaved‐Caspase3, Cleaved‐Caspase9, Bax and Bcl2) in the hepatocytes after transfected with pcDNA3.1‐VDAC1 plasmid (*n* = 3). (B) Apoptosis of the hepatocytes after transfected with the pcDNA3.1‐VDAC1 plasmid determined by flow cytometry (*n* = 3). (C) Protein expression of Nlrp3 inflammasome activation related protein (Nlrp3, Cleaved‐Caspase1, and mature Il1β) in the hepatocytes after transfected with pcDNA3.1‐VDAC1 (*n* = 3). (D) Expression of Vdac1 and apoptosis‐related proteins (Parp1, Cleaved‐Parp1, Cleaved‐Caspase3, Cleaved‐Caspase9, Bax and Bcl2) in the hepatocytes after transfected with siNC and si*vdac1* for 36 h, with or without subsequent 12 h incubation with PA (*n* = 3). (E) Apoptosis of the hepatocytes after transfected with siNC and si*vdac1* for 36 h, with or without subsequent 12 h incubation with PA determined by flow cytometry (*n* = 3). (F) Expression of Nlrp3 inflammasome activation‐related protein (Nlrp3, Cleaved‐Caspase1, and mature Il1β) in the hepatocytes after transfected with siNC and si*vdac1* for 36 h, with or without subsequent 12 h incubation with PA (*n* = 3). The results are presented as the mean ± SEM and analyzed by independent *t*‐tests. ^*^ *p* < 0.05, ^**^ *p* < 0.01, and ^***^ *p* < 0.001.

Since hepatic *vdac1* mRNA and Vdac1 protein expression were upregulated in HFD‐fed large yellow croaker (Figure S7), a VDAC1 overexpression model was established by transfecting hepatocytes with pcDNA3.1‐VDAC1 to further investigate its role in HFD‐induced apoptosis and inflammation. Overexpression of VDAC1 significantly upregulated protein expression of Cleaved‐Parp1, Cleaved‐Caspase3, Cleaved‐Caspase9, and Bax, and decreased the protein expression of anti‐apoptotic protein Bcl2 (Figure 6A). Accordingly, the flow cytometry assay showed a visibly increased apoptosis rate in the hepatocytes following VDAC1 overexpression (Figure 6B). Furthermore, VDAC1 overexpression significantly elevated the hepatocyte protein expression of inflammatory response‐related proteins, including Nlrp3, Cleaved‐Caspase1, and mature Il1β, hepatocyte Caspase1 activity and Il1β content in the culture medium of large yellow croaker (Figure 6C and Figure S8). This suggests that VDAC1 overexpression may be an important trigger for hepatocyte apoptosis and inflammatory response. Next, a *vdac1* knockdown hepatocyte model was established by transfection with si*vdac1* following PA treatment to investigate whether reduced Vdac1 expression could ameliorate excessive fatty acid‐induced apoptosis and inflammation in large yellow croaker hepatocytes. The lowest *vdac1* gene and Vdac1 protein expression levels were observed in the hepatocyte transfected with si*vdac1*#3 (Figure S9), thus si*vdac1*#3 was selected for subsequent experiments. As expected, *vdac1* knockdown markedly reversed PA‐induced upregulation of Cleaved‐Parp1, Cleaved‐Caspase3, and Cleaved‐Caspase9, as well as the PA‐induced increase in hepatocyte apoptosis rate (Figure 6D,E). However, no significant differences were observed in protein expression of Bax and Bcl2 after *vdac1* knockdown under PA treatment. The hepatocyte protein expression of Nlrp3, Cleaved‐Caspase1, and mature Il1β, hepatocyte Caspase1 activity, and Il1β content in the culture medium were also suppressed by *vdac1* knockdown under PA treatment in large yellow croaker (Figure 6F and Figure S10).

**FIGURE 7:**
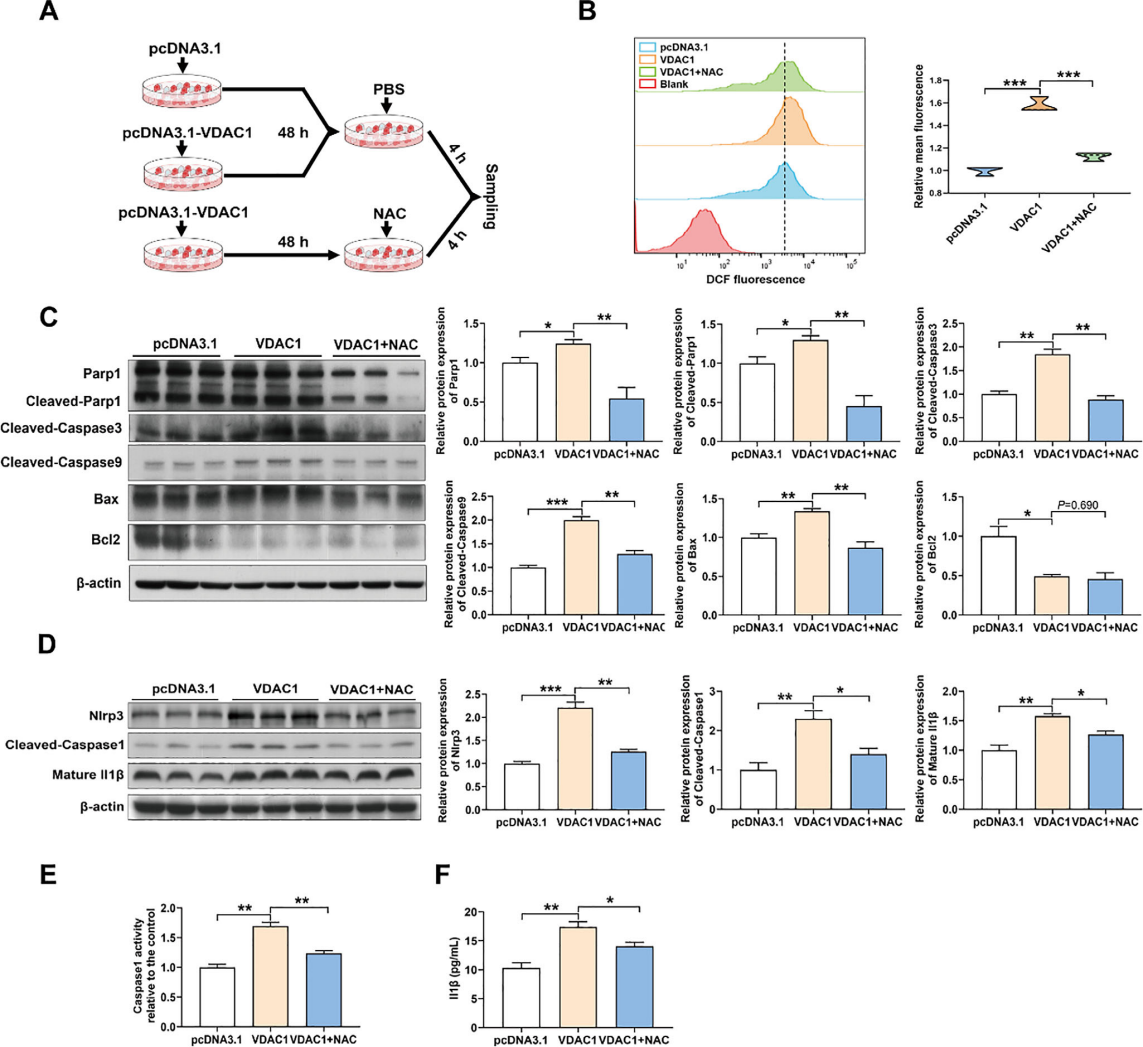
Vdac1‐ROS pathway mediates palmitic acid‐induced apoptosis and inflammation in large yellow croaker hepatocytes. (A) Experiment flowchart of VDAC1 overexpression induced apoptosis and inflammatory responses by elevating ROS levels, which was reversed by NAC, in the hepatocytes of large yellow croaker. (B) ROS levels of the large yellow croaker hepatocytes after transfected with the pcDNA3.1‐VDAC1 plasmid for 48 h, with or without subsequent 4 h incubation with NAC determined by flow cytometry (*n* = 3). (C) Protein expression of Vdac1 and apoptosis‐related protein (Parp1, Cleaved‐Parp1, Cleaved‐Caspase3, Cleaved‐Caspase9, Bax and Bcl2) (*n* = 3) and (D) Nlrp3 inflammasome activation‐related protein (Nlrp3, Cleaved‐Caspase1, and mature Il1β) in the hepatocytes after transfected with pcDNA3.1‐VDAC1 plasmid for 48 h, with or without subsequent 4 h incubation with NAC (*n* = 3). (E) Caspase1 activity in the hepatocytes after transfected with the pcDNA3.1‐VDAC1 plasmid for 48 h, with or without subsequent 4 h incubation with NAC (*n* = 3). (F) Il1β content in the culture medium of hepatocytes after transfected with the pcDNA3.1‐VDAC1 plasmid for 48 h, with or without subsequent 4 h incubation with NAC (*n* = 3). The results are presented as the mean ± SEM and analyzed by independent *t*‐tests. ^*^ *p* < 0.05, ^**^ *p* < 0.01, and ^***^ *p* < 0.001.

To determine whether the apoptosis and inflammatory responses induced by VDAC1 overexpression were mediated by ROS production, hepatocytes were treated with the ROS inhibitor N‐acetylcysteine (NAC, Figure 7A). Flow cytometry assay showed that the increased ROS production induced by VDAC1 overexpression was obviously reversed by NAC treatment (Figure 7B). Furthermore, the protein expression of apoptosis‐related proteins, including Parp1, Cleaved‐Parp1, Cleaved‐Caspase3, Cleaved‐Caspase9, and Bax, was significantly down‐regulated by NAC treatment under VDAC1 overexpression conditions, whereas Bcl2 protein expression showed no significant change (Figure 7C). Also, NAC markedly downregulated hepatocyte protein expression of Nlrp3, Cleaved‐Caspase1, and mature Il1β, hepatocyte Caspase1 activity, and Il1β content in the culture medium under VDAC1 overexpression conditions (Figure 7D–F). Overall, VDAC1 overexpression induced apoptosis and inflammatory responses through ROS production in hepatocytes of large yellow croaker.

### 2.7 *pemt* Inhibition‐Mediated Vdac1 Oligomerization Regulates Apoptosis and Inflammation in Fish

**FIGURE 8:**
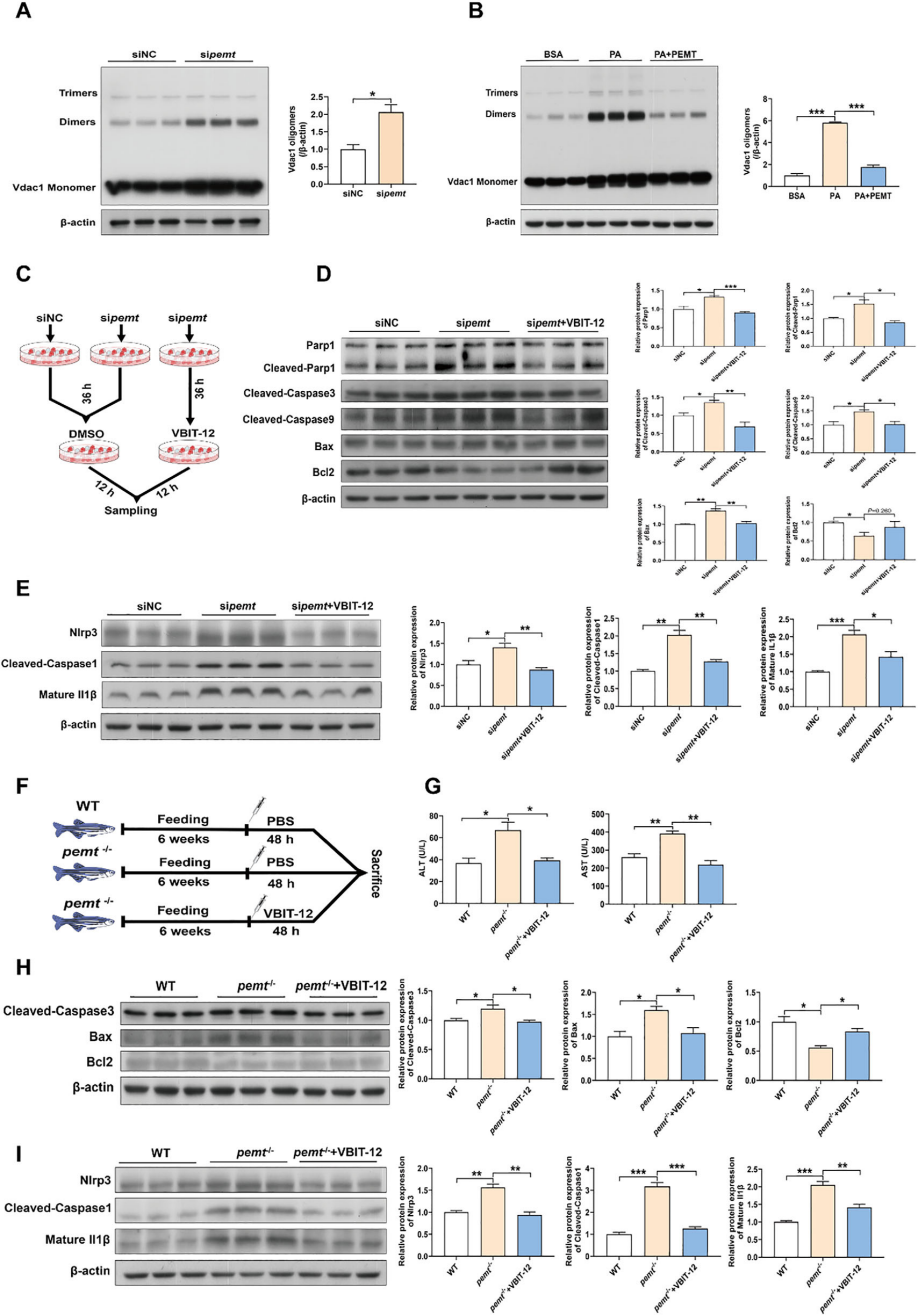
Pemt‐Vdac1 oligomerization pathway modulates apoptosis and inflammation in large yellow croaker and zebrafish. (A) Vdac1 oligomers levels of the large yellow croaker hepatocytes after transfected with si*pemt* (*n* = 3). (B) Vdac1 oligomers levels of the large yellow croaker hepatocytes after transfected with pcDNA3.1 and pcDNA3.1‐PEMT plasmid for 36 h, with or without subsequent 12 h incubation with PA (*n* = 3). (C) Experiment flowchart of Pemt inhibition induced apoptosis and inflammatory responses by increasing Vdac1 oligomerization in the hepatocytes of large yellow croaker. (D) Expression of Vdac1 and apoptosis‐related protein (Parp1, Cleaved‐Parp1, Cleaved‐Caspase3, Cleaved‐Caspase9, Bax and Bcl2) (*n* = 3) and (E) Nlrp3 inflammasome activation‐related protein (Nlrp3, Cleaved‐Caspase1, and mature Il1β) in the hepatocytes after transfected with si*pemt* for 36 h, with or without subsequent 12 h incubation with VBIT‐12 (*n* = 3). (F) Experiment flowchart of *pemt* deficiency‐induced apoptosis and inflammatory responses by increasing Vdac1 oligomerization in the liver of zebrafish. (G) Activities of ALT and AST in the serum of WT and *pemt* ^−/−^ zebrafish with subsequent 48 h injection with VBIT‐12 (*n* = 6). (H) Protein expression of apoptosis‐related protein (Cleaved‐Caspase3, Bax, and Bcl2) (*n* = 3) and (I) Nlrp3 inflammasome activation‐related protein expression (Nlrp3, Cleaved‐Caspase1, and mature Il1β) in the liver of WT and *pemt* ^−/−^ zebrafish with subsequent 48 h injection with VBIT‐12 (*n* = 3). The results are presented as the mean ± SEM and analyzed by independent *t*‐tests. ^*^ *p* < 0.05, ^**^ *p* < 0.01, and ^***^ *p* < 0.001.

VDAC1 oligomerization facilitates the formation of large pores in the outer mitochondrial membrane, triggering apoptosis and inflammatory responses [51]. The increased oligomerization of Vdac1 was observed in hepatocytes following *pemt* knockdown (Figure 8A). Meanwhile, *pemt* knockdown aggravated PA‐induced increase in protein expression and oligomerization of Vdac1 (Figure S11). Notably, PEMT overexpression effectively reversed the increase in Vdac1 oligomerization induced by PA treatment (Figure 8B), suggesting the regulatory role of Pemt in Vdac1 oligomerization status. To establish whether *pemt* deficiency promotes apoptosis and inflammatory response through Vdac1 oligomerization, the Vdac1 oligomerization inhibitor VBIT‐12 was incubated with large yellow croaker hepatocytes under *pemt* knockdown conditions (Figure 8C). The PA‐induced increase in both oligomerization and protein expression of Vdac1 was attenuated by VBIT‐12 in large yellow croaker hepatocytes (Figure S12). The hepatocyte protein expression levels of Parp1, Cleaved‐Parp1, Cleaved‐Caspase3, Cleaved‐Caspase9, Bax, Nlrp3, Cleaved‐Caspase1, and mature Il1β, as well as hepatocyte Caspase1 activity and Il1β content in the culture medium, were significantly decreased when large yellow croaker hepatocytes were treated with VBIT‐12 under *pemt* knockdown conditions (Figure 8D,E and Figure S13A,B). These results suggest that *pemt* knockdown induced apoptosis and inflammatory responses through Vdac1 oligomerization. To further confirm the role of the Pemt‐Vdac1 oligomerization pathway in liver injury, hepatocyte apoptosis, and inflammatory response in fish, *pemt* ^−/−^ zebrafish were treated with VBIT‐12 via intraperitoneal injection (Figure 8F). The activities of ALT and AST were significantly decreased in the serum of *pemt* ^−/−^ zebrafish under VBIT‐12 administration compared with those of *pemt* ^−/−^ zebrafish under PBS administration, suggesting that Vdac1 oligomerization mediated *pemt* deficiency‐induced liver injury (Figure 8G). Meanwhile, VBIT‐12 treatment in *pemt* ^−/−^ zebrafish also significantly downregulated the protein expression of Cleaved‐Caspase 3, Bax, Nlrp3, Cleaved‐Caspase1, and mature Il1β, and upregulated the protein expression of Bcl2 (Figure 8H,I). Meanwhile, the hepatocyte Caspase1 activity and serum Il1β content were significantly decreased by VBIT‐12 treatment in *pemt* ^−/−^ zebrafish (Figure S13C,D). These collective findings demonstrate that *pemt* deficiency‐induced liver injury in fish was mediated by Vdac1 oligomerization.

## 3 Discussion

Accumulating evidence demonstrates that HFD‐induced mitochondrial damage serves as a pivotal trigger for apoptosis and NLRP3 inflammasome activation, ultimately leading to liver injury, and this phenomenon is conserved across vertebrates from mammals to teleost fish [25, 27, 52, 53]. As the key structural lipid in cellular and organelle membranes, PC is essential for maintaining mitochondrial functional homeostasis and regulating diverse cellular physiological processes [54]. PEMT, an important PC synthase localized in MAM, emerges as a potential regulator of mitochondrial structural and functional homeostasis. However, the precise mechanism by which PEMT contributes to mitochondrial dysfunction‐induced liver injury remains unclear. Clinical data reveal reduced gene expression of *PEMT* in MASH patients [18], suggesting its pathophysiological relevance. In the present study, we successfully established an HFD‐induced model of mitochondrial damage and liver injury in large yellow croaker, observing concomitant reductions in hepatic PC content and Pemt expression. These findings confirm the conservation of HFD‐induced suppression of PC synthesis and subsequent liver injury from mammals to teleost fish. Notably, *pemt* knockdown recapitulated HFD‐induced hepatocyte apoptosis and inflammation, whereas overexpression attenuated these effects when induced by PA. These results were further corroborated in *pemt* ^−/−^ zebrafish, which exhibited exacerbated hepatic apoptosis and inflammatory responses. Supporting our findings, a previous study reported that *Pemt* ^−/−^ mice fed high‐fat‐high‐sucrose diets exhibited enhanced apoptosis of hepatocytes [55], while HFD‐fed mice with *Pemt* deficiency showed increased hepatic oxidative stress and inflammatory responses within 10 weeks [56]. Together, these results reveal a conserved mechanism whereby HFD drives hepatocytes' apoptosis and inflammation through Pemt suppression, ultimately precipitating liver injury.

Mitochondria have emerged as central regulators of cellular inflammatory responses, particularly NLRP inflammasome activation and apoptosis [57]. Given the essential role of PC in mitochondrial homeostasis [54], the influence of Pemt on mitochondrial function was investigated. A previous work revealed that *Pemt* deficiency promoted the reduction of complexes I, III, and IV of the electron transport system in the brown adipose tissue of HFD‐fed mice [15], suggesting a direct link between *Pemt* deficiency and mitochondrial dysfunction. The MMP, a critical indicator of mitochondrial integrity, is known to contribute significantly to the pathogenesis of metabolic dysregulations [58]. Similarly, the present study found that the MMP was markedly decreased after *pemt* knockdown in large yellow croaker hepatocytes, indicating that decreasing *pemt* expression can damage the mitochondrial integrity. Previous studies have revealed that mitochondrial homeostasis is maintained by mitochondrial quality control systems, including mitochondrial biogenesis and fusion [59]. Mitochondrial regeneration involves the replacement of dysfunctional organelles through coordinated mechanisms involving key regulators such as PGC1α, nuclear respiratory factor 1 (NRF1), and ESRRα, while the fusion factors such as mitofusins and OPA1 work in coordination with biogenesis to promote maturation of mitochondria and functional homeostasis [60]. Intriguingly, *pemt* knockdown suppressed both mitochondrial biogenesis and fusion in hepatocytes, suggesting that Pemt‐mediated PC synthesis may be essential for mitochondrial renewal. Furthermore, elevated ROS generation following *pemt* knockdown further confirmed mitochondrial dysfunction, while PEMT overexpression reversed these results under PA treatment. These observations were further validated in *pemt* ^−/−^ zebrafish model. For example, *pemt* ^−/−^ zebrafish showed increased ROS generation and mitochondrial structural damage compared with those in WT zebrafish. Notably, *pemt* deficiency exacerbates disturbed mitochondrial biogenesis and fusion processes and significantly reduces cardiolipin content in the liver of fish. Cardiolipin, a mitochondria‐specific phospholipid, is essential for maintaining cristae structure and facilitating metabolic reactions within mitochondrial membranes [49]. Similarly, a previous study reported that HFD‐fed *Pemt* knockout mice exhibited decreased mitochondrial cardiolipin content, a signature phospholipid of mitochondrial membranes, and exhibited cold‐intolerant [15]. Therefore, these results highlight the important role of Pemt in excessive fatty acids‐induced mitochondrial damage in fish.

To further investigate the molecular mechanisms by which Pemt‐mediated HFD‐induced mitochondrial damage and liver injury, we identified Vdac1 as a mitochondrial outer membrane protein interacting with Pemt. VDAC1, a key mitochondrial gatekeeper, regulates mitochondrial homeostasis and cell fate [61], and is well‐established in apoptosis and inflammatory responses [62, 63]. Overexpression of VDAC1 induces apoptotic cell death and inflammation across various cell types [64, 65]. Consistent with these findings, the present study revealed that overexpression of VDAC1 significantly induced apoptosis and Nlrp3 inflammasome activation‐mediated inflammation in large yellow croaker hepatocytes. Conversely, *vdac1* inhibition attenuated PA‐induced Nlrp3 inflammasome activation and apoptosis in the hepatocytes. Similarly, a previous study also showed that sennoside A reversed HFD‐induced mitochondrial damage and hepatic steatosis by decreasing VDAC1 protein expression [62]. The downregulation of VDAC1 markedly reduced the apoptosis in endothelial cells [66]. These results suggest a conserved role for VDAC1 in excessive fatty acid‐induced apoptosis and inflammatory response. Mitochondrial damage‐induced ROS elevation serves as a key initiator of caspase cascade‐mediated apoptosis and NLRP3 inflammasome activation [25, 67]. Notably, VDAC1 overexpression‐induced oligomerization is closely associated with ROS overproduction [68, 69], and the present study also showed that VDAC1 overexpression markedly increased hepatocyte ROS levels. Using ROS scavengers, the present study demonstrated that excess fatty acids may induce apoptosis and inflammation by upregulating the VDAC1‐ROS pathway.

VDAC1 oligomerization represents a therapeutic target for multiple diseases [70], as it forms large mitochondria outer membrane channels that facilitate the translocation of pro‐apoptotic proteins and ROS from the mitochondria intermembrane space to the cytoplasm, triggering cell death and inflammation [61, 71]. Our data identified that *pemt* knockdown aggravated the PA‐induced increase in Vdac1 protein expression and oligomerization in hepatocytes. Meanwhile, PEMT overexpression significantly reversed PA‐induced Vdac1 oligomerization in hepatocytes. Collectively, these findings suggest that fatty acid overload inhibits Pemt expression, thereby promoting Vdac1 upregulation and shifting the protein from a monomeric to an oligomeric state. A previous study demonstrated that apoptosis and inflammation were prevented by VBIT‐12, an oligomerization inhibitor of VDAC1, in mice with ulcerative colitis [51]. Similarly, both the hepatocyte *pemt* knockdown model and the *pemt* knockout zebrafish model found that the apoptotic and inflammatory responses induced by *pemt* deficiency were also attenuated by VBIT‐12. These findings further suggest that *pemt* deficiency exacerbates hepatocyte injury through the Vdac1 oligomerization pathway.

## 4 Conclusion

**FIGURE 9:**
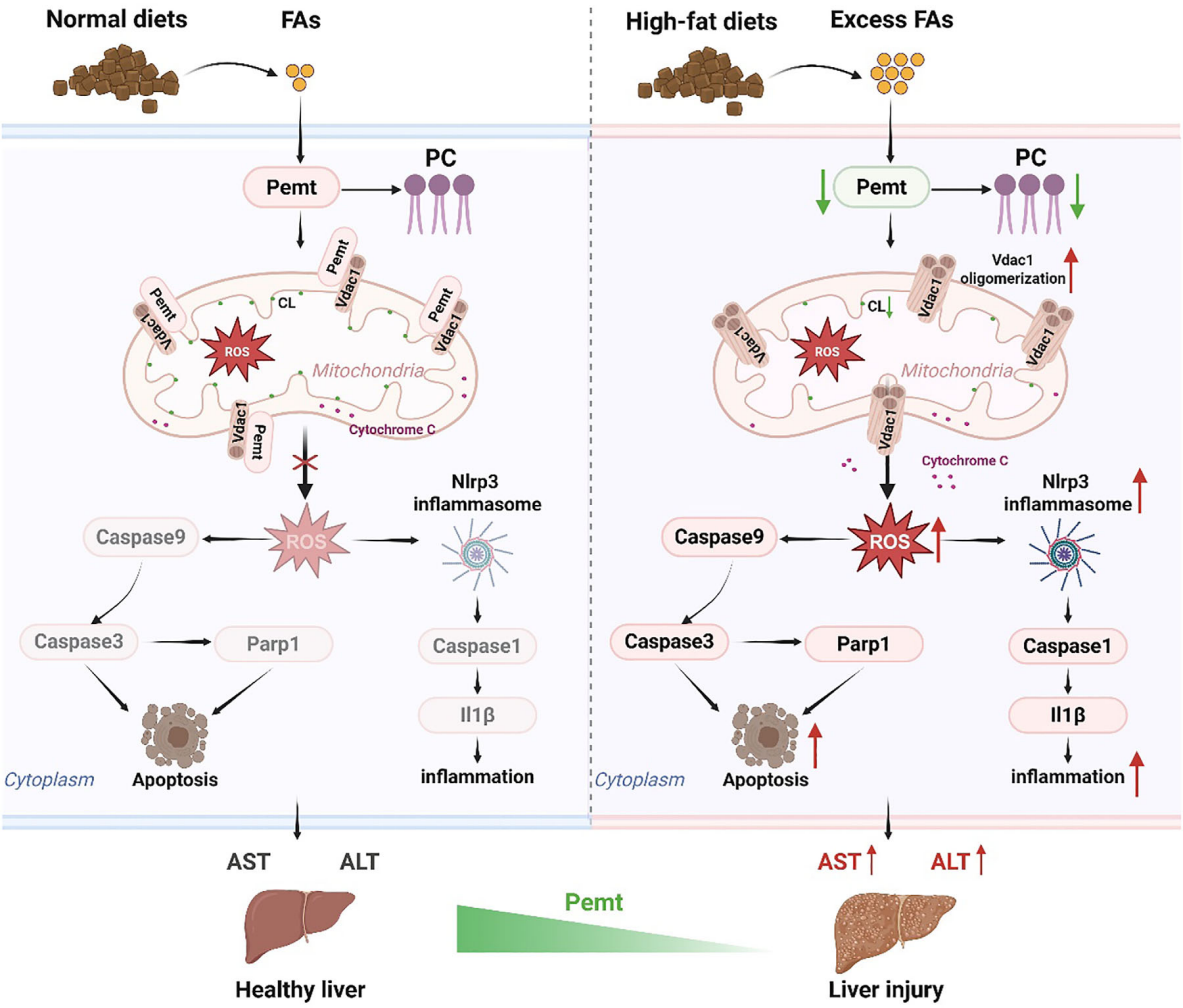
Schematic model: HFD‐induced Pemt inhibition exacerbates mitochondrial dysfunction by promoting Vdac1 oligomerization, thereby amplifying apoptosis and inflammatory responses.

In conclusion, our findings elucidate a crucial regulatory mechanism in which HFD‐induced hepatic Pemt suppression exacerbates mitochondrial dysfunction by promoting Vdac1 oligomerization, thereby amplifying apoptosis and inflammatory responses (Figure 9). These findings highlight the Pemt‐Vdac1 oligomerization axis as a promising target for mitigating mitochondrial dysfunction‐associated liver injury in vertebrates.

## 5 Experimental Section

### 5.1 Animal Experiments

All animal experiments and care were performed following protocols approved by Ocean University of China's Institutional Animal Care and Use Committee and in compliance with the Management Rule of Laboratory Animals (State Council Order No. 676, revised March 1, 2017).

Mixed‐sex large yellow croakers were sourced from the Aquatic Fingerlings Limited Company (Xiangshan Harbour, Ningbo, China). Two isonitrogenous experimental diets containing 12% (used as Con) and 18% (used as HFD) crude lipid were formulated for use in the feeding trial of large yellow croaker (Table S1). Prior to the formal feeding trial of large yellow croaker, fish were hand‐fed the control diet twice daily (06:00 and 18:00) to acclimatize the experimental diets and conditions for 2 weeks. Then, fish were randomly divided into two groups and maintained in floating sea cages (1.0 × 1.0 × 2.0 m) under conditions of 26°C–30°C, 5.5–7.0 mg/L dissolved oxygen content, 7.0–7.3 pH, and 29.0‰–32.0‰ salinity. Prior to sampling, fish received MS‐222 (1:10 000 w/v; Sigma–Aldrich, USA) for anesthesia, and liver and serum from nine fish each cage were sampled, frozen in liquid nitrogen, and maintained at ‐80°C until analysis.

Zebrafish broodstock (6‐month old) were purchased from the Chinese National Zebrafish Resource Center (Wuhan, China). Embryos were obtained through natural spawning and reared in a plastic tank (11.5 × 28.0 × 16.0 cm, L × W × H) at 28°C. A diet of freshly hatched brine shrimp eggs was administered twice daily. To examine the effect of *pemt* knockout on apoptosis and inflammation, the WT and *pemt* ^−/−^ zebrafish (4‐month‐old) were fed a formulated diet (Table S2) containing 10% crude lipid for 6 weeks. To determine the role of VBIT‐12 in *pemt* knockout‐induced apoptosis and inflammation, the WT and *pemt* ^−/−^ zebrafish was fed a formulated diet for 6 weeks, followed by intraperitoneal injection of phosphate‐buffered saline (PBS) or VBIT‐12 (20 mg/kg) into the zebrafish for 48 h. All zebrafish were reared at 28°C in a recirculating aquaculture system under a 14/10 h light/dark cycle. At the end of the experiment, the liver and serum of fish were sampled, frozen in liquid nitrogen, and stored at ‐80°C until use.

The *pemt* ^−/−^ zebrafish line was generated using the CRISPR/Cas9 tool. Detailed protocols for the generation of *pemt* knockout zebrafish are provided in the Supplemental Methods section. The resultant F3 *pemt* ^−/−^ zebrafish and WT zebrafish were used for subsequent feeding trials and analyzed for ROS staining (15 dpf) using the fluorescent probe DCFH‐DA (10 µm).

### 5.2 Plasmid Construction

The plasmids were generated using the recombinant DNA method. For subcellular localization and co‐localization analyses, the open reading frames (ORFs) of *pemt* or *vdac1* gene were cloned into the pcDNA3.1‐GFP or pcDNA3.1‐RFP vector, respectively. For overexpression studies, both ORFs were inserted into the pcDNA3.1‐Basic vector. For co‐immunoprecipitation experiments, the sequences of Flag‐tagged *pemt* and Ha‐tagged *vdac1* were constructed into the pcDNA3.1‐Basic vector. For the bimolecular fluorescence complementation assay, the ORFs of the *pemt* or *vdac1* gene were constructed into pBiFC‐VN173 or pBiFC‐VC155 empty vectors, respectively. The primers used for plasmid construction are listed in Table S5.

### 5.3 Cell Culture and Treatment

Primary hepatocytes were isolated from large yellow croaker liver tissue via enzymatic digestion with 0.25% trypsin. Hepatocytes were cultured in DMEM/F12 medium (Biological Industries, Israel) containing 100 U/mL penicillin, 100 µg/mL streptomycin, and 15% fetal bovine serum (FBS). HEK293T cells were grown in high‐glucose DMEM (Biological Industries, Israel) containing 1% antibiotics (Solarbio, China) and 10% FBS at 37°C under 5% CO_2_. When the hepatocytes of large yellow croakers reached the logarithmic phase, they (1) were treated with 400 µm of PA for 12 h; (2) were transfected with si*pemt* (sequences are shown in Table S3) using electroporation for 24 h (For gene expression analysis) or 48 h (For protein expression analysis); (3) were transfected with pcDNA3.1‐PEMT plasmid or si*pemt* for 12 h (For gene expression analysis) or 36 h (For analyses of protein expression, hepatocyte Caspase1 activity and culture medium Il1β content) followed by PA treatment for 12 h; (4) were transfected with pcDNA3.1‐VDAC1 plasmids for 48 h, with or without subsequent 4 h incubation with NAC (2.5 mm, Sigma, USA); (5) were transfected with si*vdac1* (sequences are shown in Table S3) using electroporation for 24 h (For gene expression analysis) or 48 h (For protein expression analysis); (6) were transfected with *vdac1* siRNA using electroporation for 36 h followed by PA treatment for 12 h; (7) were treated with 0, 5, 10, 20 µm VBIT‐12 (MedChemExpress, USA) for 36 h followed by PA treatment for 12 h; (8) were transfected with si*pemt* using electroporation for 36 h followed by VBIT‐12 (10 µm, MedChemExpress, USA) treatment for 12 h in the presence of PA.

### 5.4 Histological and Ultrastructural Analysis

Liver specimens underwent 24 h immersion fixation in 4% paraformaldehyde at 4°C, followed by graded ethanol dehydration. After paraffin embedding, tissues were sectioned at 6 µm thickness with a microtome according to established protocols [72]. Resulting sections received hematoxylin and eosin (HE) staining prior to microscopic examination using an Olympus BX53 system (Olympus, Japan).

For ultrastructural analysis, liver tissues from large yellow croaker and zebrafish were fixed in 2.5% glutaraldehyde (Solarbio, China). After fixation, samples were post‐fixed in 1% osmium tetroxide, dehydrated in a graded acetone series (10%–100%), followed by staining with uranyl acetate and lead citrate. Ultrastructural analysis was conducted using a TEM (JEM‐1200, Japan).

### 5.5 Biochemical Analysis

Hepatic TG and TC levels and the activities of ALT and AST in the serum were determined using commercial enzyme assay kits (JianCheng Bioengineering Institute, Nanjing, China) according to the instructions. The T‐AOC, GSH content, T‐SOD activity, and MDA content were also determined using commercial enzyme assay kits (JianCheng Bioengineering Institute, Nanjing, China).

### 5.6 Lipid Class Composition, PC and PE Contents Analysis

Total lipids were extracted from liver tissues using chloroform/methanol (2:1, v/v), and neutral and polar lipid classes in the liver were separated by thin‐layer chromatography through the mobile phase reported in a previous study [73]. The contents of PC and phosphatidylethanolamine (PE) were examined with enzyme‐linked immunosorbent assay kits (Fankew, Shanghai, China).

### 5.7 Detection of ROS Production

The ROS contents of large yellow croaker hepatocytes and zebrafish were quantified utilizing a ROS detection kit (Beyotime, Shanghai, China) in accordance with the manufacturer's protocol. The fluorescent probe DCFH‐DA was prepared as a 10 µm working solution in serum‐free DMEM/F12 medium (for hepatocytes) or water (for zebrafish). Samples were washed with PBS or water and subsequently exposed to the working solution for 30 min at 28°C in the dark. After three washes with serum‐free medium or water to remove unbound probe, fluorescence intensity was measured using a flow cytometer (BD Biosciences, USA) or a fluorescent microscope (BioTek, USA).

### 5.8 Detection of Apoptosis Rate

Cell apoptosis was quantified with an Annexin V‐FITC/PI Apoptosis Detection Kit (C1062, Beyotime, China). Following EDTA‐free trypsin harvesting from six‐well plates, cells underwent PBS washing and centrifugation (800 × g, 5 min). Pellets were resuspended in 195 µL binding buffer, then stained with 10 µL propidium iodide and 5 µL Annexin V‐FITC. After 20 min dark incubation at room temperature, samples were immediately subjected to flow cytometric analysis (BD Biosciences, USA), with data processed using FlowJo V10. For zebrafish liver sections, apoptosis was assessed via TUNEL staining. Post‐fixation in 4% paraformaldehyde and Triton X‐100 permeabilization, sections were incubated with TUNEL reaction mixture (containing fluorescein‐dUTP and TdT enzyme). Nuclei were counterstained with DAPI, and apoptotic cells were visualized by fluorescence microscopy (Nikon, Japan).

### 5.9 RNA Extraction and RT‐qPCR

Total RNA was isolated from liver tissue or hepatocytes using the TRIzol Reagent (Takara, Japan) and quantified using the Nanodrop 2000 spectrophotometer (Thermo Fisher Scientific, USA). For cDNA synthesis, 1 µg of total RNA was reverse‐transcribed using the PrimeScript RT Reagent Kit (Takara, Japan) in accordance with the kit protocol. Primers were designed using Primer Premier 5.0 software according to previously published sequences in NCBI (Table S4). RT‐qPCR was performed using the ChamQ Universal SYBR qPCR Master Mix (Vazyme, China), and gene expression levels were calculated using the 2^−ΔΔCT^ method [74]. Relative quantification was normalized to 18S rRNA as the endogenous reference gene.

### 5.10 Western Blotting

Protein extraction from liver tissues and hepatocytes utilized ice‐cold RIPA lysis buffer (Solarbio, China) containing protease and phosphatase inhibitors. Protein concentrations were determined with a BCA Assay Kit (Beyotime, China). Following SDS‐PAGE separation, proteins were transferred onto 0.45 µm polyvinylidene fluoride membranes (Millipore, USA). Membranes were subsequently blocked with 5% non‐fat milk for 2 h, followed by overnight incubation at 4°C with anti‐PARP1 (66520, Protentech, USA), anti‐CASPASE3 (19677, Protentech, USA), anti‐ CASPASE9 (9508, CST, USA), anti‐BAX (50599, Protentech, USA), anti‐BCL2 (4223, CST, USA), anti‐NLRP3 (preparation by Genscript, China), anti‐CASPASE1 (preparation by Genscript, China), anti‐IL1β (preparation by Genscript, China), anti‐PEMT (preparation by Genscript, China), anti‐β‐actin (AF7018, Affinity, USA), anti‐VDAC1 (R1307‐1, HUABIO, Hangzhou, China), anti‐DYKDDDDK Tag (14793, CST, USA), anti‐HA Tag (3724, CST, USA) primary antibodies. Afterward, membranes were incubated with HRP secondary antibodies for 1 h, followed by imaging with BeyoECL Reagent (Beyotime, Shanghai, China). The protein band densities were analyzed by ImageJ 1.63 software.

### 5.11 Mitochondrial Membrane Potential Assay

Mitochondrial membrane potential (∆Ψm) was assessed using the fluorescent probe JC‐1 (Beyotime, China). Large yellow croaker hepatocytes were seeded in six‐well plates and incubated with JC‐1 staining working solution for a duration of 20 min at 28°C under dark conditions. After incubation, hepatocytes were washed twice with ice‐cold staining buffer. Fluorescence imaging was performed using a laser confocal microscope (Leica, Germany) with dual‐channel detection (excitation/emission: 488/530 nm for monomeric green; 561/595 nm for aggregate red).

### 5.12 Subcellular Localization

The HEK 293T cells were cultured in an atmosphere containing 5% CO_2_ at 37°C according to previously established protocols [75]. For subcellular localization studies, cells were transiently transfected with either pcDNA3.1‐PEMT‐GFP or pcDNA3.1‐VDAC1‐RFP plasmids using EZ Trans Cell transfection reagent (Life iLab, Shanghai, China). Then, cells were washed with PBS and fixed in 4% paraformaldehyde for 15 min at room‐temperature. Mitochondria were stained with fluorescent mitochondrial indicator MitoLite Orange (22676, AAT Bioquest, USA), and endoplasmic reticulum (ER) was stained with ER‐Tracker (C1042, Beyotime, Shanghai, China). Nuclear staining was performed with DAPI (Beyotime, Shanghai, China), and the fluorescence signal was determined using a laser confocal microscope (Leica, Germany).

### 5.13 Lipidomic Analysis

Lipidomic profiling employed ultra‐high‐performance liquid chromatography coupled to high‐resolution mass spectrometry (UHPLC‐HRMS; Thermo Scientific, USA) as described previously [76]. Briefly, the total lipids were extracted from liver tissues using chloroform/methanol (1:1, v/v) solution. After ultrasonication (50 kHz, 3 min), the mixture was centrifuged at 12 000 × *g* for 15 min at 4°C. Following collection of the organic phase, chromatographic separation was conducted on a Waters ACQUITY UPLC BEH C18 column (2.1 × 100 mm, 1.7 µm) employing a binary solvent system (Mobile phase A: aqueous solution containing 25 mmol/L ammonium acetate and 25 mmol/L aqueous ammonia; Mobile phase B: acetonitrile). Mass spectrometric analysis operated in dual ionization modes with optimized parameters: positive mode spray voltage at 3.5 kV and negative mode at 2.8 kV, capillary temperature maintained at 320°C, sheath gas flow 45 arbitrary units, and auxiliary gas flow 15 arbitrary units. Raw data were analyzed using LipidSearch 4.2 software for peak alignment, lipid annotation (referenced against the LIPID MAPS database), and normalization. Differential lipids were identified through orthogonal partial least squares discriminant analysis (OPLS‐DA) with thresholds of variable importance in projection (VIP) > 1.0 and *P* value < 0.05.

### 5.14 GST Pull‐Down and LC‐MS/MS Analysis

To identify interacting partners of Pemt in large yellow croaker, the full‐length *pemt* coding sequence was cloned into pGEX‐4T2 for GST‐fusion protein expression. GST‐PEMT and GST control proteins were affinity‐purified using Glutathione Sepharose 4B beads. Total protein lysates were incubated with immobilized beads at 4°C for 4 h. After five washes, bound proteins were eluted under denaturing conditions, reduced/alkylated (DTT/IAA), and digested with trypsin (Promega). Peptides were analyzed by LC‐MS/MS (Gene Create, China).

### 5.15 Co‐Immunoprecipitation

HEK293T cells were cultured overnight and transfected with PCS2‐PEMT‐Flag and PCS2‐VDAC1‐Ha plasmids using EZ Trans Cell transfection reagent (Life iLab, Shanghai, China). After 48 h of transfection, cells were harvested by centrifugation (800 × *g*, 5 min, 4°C) and lysed in NP‐40 buffer (Beyotime, Shanghai, China) containing protease inhibitors. Subsequently, the lysates were incubated with IgG‐beads (Sigma–Aldrich, USA), PierceANTI‐HA agarose (Thermo Scientific, USA) and ANTI‐FLAGM2 affinity gel (Sigma–Aldrich, USA) at 4°C overnight. Then, the fusion protein was washed thrice and eluted with Flag or HA peptides (APExBIO, USA) for 30 min at 4°C. Eluates were analyzed by SDS‐PAGE followed by immunoblotting.

### 5.16 Bimolecular Fluorescence Complementation (BiFC) Assay

HEK293T cells were cultured in glass bottom dishes (ThermoFisher, USA) overnight, and cells (1) were co‐transfected with the pcDNA3.1‐VDAC1‐RFP plasmid and empty BiFC vectors (pBiFC‐VN173 and pBiFC‐VC155); (2) were co‐transfected with the pBiFC‐VN173‐PEMT, pBiFC‐VC155‐VDAC1, and pcDNA3.1‐VDAC1‐RFP plasmids using EZ Trans reagent (Life iLab, Shanghai, China). At 24 h post‐transfection, nuclear was stained with DAPI (Beyotime, Shanghai, China). Fluorescent signals were captured using a laser confocal microscope (Leica, Germany).

### 5.17 Vdac1 Cross‐Linking Assay

The hepatocytes of large yellow croakers were washed with ice‐cold PBS and harvested by mechanical scraping. For chemical crosslinking, cell pellets were resuspended in PBS (pH 7.4) and treated with 0.5 mm ethylene glycol bis (succinimidyl succinate) (EGS; Thermo Fisher Scientific) for 30 min at room‐temperature. The reaction was quenched by adding 1.5 M Tris HCl (pH 7.8, final 20 mm) for 5 min. Cells were then centrifugated at 10 000 × *g* for 5 min (4°C), and pellets were lysed in ice‐cold NP‐40 buffer (Beyotime, Shanghai, China) supplemented with protease inhibitors. Following separation by SDS‐PAGE, proteins were transferred and immunoblotted with anti‐VDAC1 antibody (R1307‐1, HUABIO, Hangzhou, China).

### 5.18 Statistical Analysis

Results are presented as mean ± standard error of the mean (SEM). Statistical differences were assessed using a two‐tailed Student's *t*‐test in SPSS 20.0 (IBM Corporation). A *p* value of ^*^ < 0.05, ^**^ < 0.01, or ^***^ < 0.001 was considered statistically significant.

## Author Contributions

X. B. and X. C. performed experiments and analyzed data. X. Y., B. L., and Z. C. reared the experimental fish. Q. A., X. B., X. C., W. L., Z. Z., T. H., J. D., and Y. L. conceptualized and designed the research. Q. A., K. M., and X. B. gained the research funding. X. B. drafted, and all other authors revised the manuscript.

## Conflicts of Interest

The authors declare no conflicts of interest.

## Supporting information




**Supporting File**: advs76060‐sup‐0001‐SuppMat.docx.

## Data Availability

The data that support the findings of this study are available from the corresponding author upon reasonable request.
