# Supplementary material for: Pemt Inhibition‐Mediated Vdac1 Oligomerization Regulates Mitochondrial Dysfunction, Apoptosis, and Inflammation in High‐Fat Diet‐Derived Liver Injury
Source: Adv Sci (Weinh). 2026 Jul 2:e76060. Online ahead of print. doi: 10.1002/advs.76060 (PMC13336821; doi:10.1002/advs.76060)
Supplement: Supplementary file 1 — Supporting File: advs76060‐sup‐0001‐SuppMat.docx. [file ADVS-9999-e76060-s001.docx]

Supporting Information

**Pemt Inhibition-mediated Vdac1 Oligomerization Regulates Mitochondrial Dysfunction, Apoptosis and Inflammation in High-fat Diet-derived Liver Injury**

Xianyong Bu^1#^, Xiufei Cao^1#^, Xin Yao^1^, Baolin Li^1^, Wencong Lai^1^, Zengqi Zhao^1^, Tingting Hao^1^, Zhiwei Chen^1^, Jianlong Du^1^, Yueru Li^1^, Kangsen Mai^1^, Qinghui Ai^1^*

**Supplemental Methods**

The *pemt* knockout zebrafish establishment

The CRISPR/Cas9 target sites were designed by using the online tool (ZiFiT Targeter Version 4.2, http://zifit.partners.org/ZiFiT/). The Cas9 messenger RNAs were generated by in vitro transcription from pCS2-nCas9n by using the mMESSAGE mMACHINE T3 kit (Invitrogen, CA). Double strand DNA for specific gRNA synthesis was amplified by PCR from pT7-gRNA. The target single-guide RNAs (sgRNAs) was synthesized using TranscriptAid™ T7 High Yield Transcription Kit (Thermo Scientific, USA). The Cas9 messenger RNA and sgRNA were co-microinjected into one-cell-stage embryos using Picoliter Microinjector PLI-100A (Warner Instruments, Hamden, USA). The F0 founders carrying mosaic mutations were identified by high-resolution melt analysis and heteroduplex motility assay and mated with wild type (WT) zebrafish to produce heterozygous F1 offsprings. The *pemt* heterozygotes were allowed to self-cross to obtain the *pemt*^-/-^ homozygous mutants (F2), resulting into deletion of -2 bp and insertion of +3 bp fragment on the target sites. The F3 embryos of WT and mutants were obtained from F2 adult fish through natural mating and kept in Petri dishes with static water at 28 ℃. The zebrafish larvae were transferred into an aquarium with clear water after at 5-6 dpf and fed live paramecia two times per day. After 15 dpf-old of zebrafish, they were maintained at 28 ℃ in a recirculating aquaculture system equipped with a mechanical filter and an aquarium heater and kept under a 14 h/10 h light/dark cycle.

**Supplemental Tables**

**Table S1 Formulation and proximate composition of the experimental diets (% dry weight)**

|  | Dietary lipid levels (%) | |
| --- | --- | --- |
| Ingredients | Con (12%) | HFD (18%) |
| White fish meal^a^ | 36.00 | 36.00 |
| Wheat gluten^a^ | 15.00 | 15.00 |
| Soy protein concentrate^a^ | 11.00 | 11.00 |
| Fish oil^a^ | 7.50 | 13.50 |
| Lecithin^a^ | 1.50 | 1.50 |
| Tapioca starch^a^ | 19.00 | 19.00 |
| Vitamin premix^b^ | 0.40 | 0.40 |
| Vitamin C phosphate^a^ | 0.10 | 0.10 |
| Mineral premix^b^ | 0.50 | 0.50 |
| Monocalcium phosphate^a^ | 0.90 | 0.90 |
| Ethoxyquin | 0.10 | 0.10 |
| Microcrystalline cellulose^a^ | 7.92 | 1.92 |
| Choline chloride^c^ | 0.08 | 0.08 |
| Total | 100 | 100 |
| Proximate composition (%) |  |  |
| Crude protein | 43.11 | 43.41 |
| Crude lipid | 12.67 | 18.08 |

^a^ All of these ingredients were supplied by Guangdong Yuehai Feeds Group, China.

^b^ The mixture of vitamin and mineral according to Chen et al. (2021).

^c^ Choline chloride (99%) was bought from Shanghai Aladdin Bio-Chem Technology Co.LTD, China.

**Table S2 Formulation and proximate composition of the experimental diets (% dry weight)**

| Ingredients | Diet |
| --- | --- |
| Casein^a^ | 32.00 |
| Gelatin^a^ | 8.00 |
| Corn starch^a^ | 30.00 |
| Soybean oil^a^ | 10.00 |
| Vitamin premix^b^ | 1.00 |
| Mineral premix^b^ | 3.00 |
| Monocalcium phosphate | 1.00 |
| butylated hydroxytoluene | 0.02 |
| Dimethyl-β-propiothetin | 0.10 |
| Microcrystalline cellulose^a^ | 10.38 |
| Carboxymethyl cellulose^a^ | 4.00 |
| Choline chloride^c^ | 0.50 |
| Total | 100 |
| Proximate composition (%) |  |
| Crude protein | 41.14 |
| Crude lipid | 10.50 |

^a^ All of these ingredients were supplied by Great Seven Biotechnology Co., Ltd., China.

^b^ The mixture of vitamin and mineral according to Chen et al. (2021).

^c^ Choline chloride (99%) was bought from Shanghai Aladdin Bio-Chem Technology Co.LTD, China.

**Table S3 Oligonucleotides sequences of siRNAs**

| Primer | Sequences (5’-3’) |
| --- | --- |
| *pemt*-siRNA1#-F | GACUCAACAACGUCGAUUATT |
| *pemt*-siRNA1#-R | UAAUCGACGUUGUUGAGUCTT |
| *pemt*-siRNA2#-F | GAACCUUCUUGGGUGAUUATT |
| *pemt*-siRNA2#-R | UAAUCACCCAAGAAGGUUCTT |
| *pemt*-siRNA3#-F | GGCGAUAAGAUUUGAGGGATT |
| *pemt*-siRNA3#-R | UCCCUCAAAUCUUAUCGCCTT |
| *vdac1*-siRNA1#-F | GAUGUCAACUACGAUAUCATT |
| *vdac1*-siRNA1#-R | UGAUAUCGUAGUUGACAUCTT |
| *vdac1*-siRNA2#-F | GCUGCAUACAAAUGUAAAUTT |
| *vdac1*-siRNA2#-R | AUUUACAUUUGUAUGCAGCTT |
| *vdac1*-siRNA3#-F | GCUACACUCAGACUCUGAATT |
| *vdac1*-siRNA3#-R | UUCAGAGUCUGAGUGUAGCTT |

*pemt*, phosphatidylethanolamine N-methyltransferase; *vdac1*, voltage dependent anion channel 1; F, forward; R, reverse.

**Table S4 Primer sequences of genes used for real-time PCR**

| Primer | Sequences (5’-3’) |
| --- | --- |
| For large yellow croaker |  |
| *18s rRNA*-F | GTTCTTAGTTGGTGGAGCG |
| *18s rRNA*-R | GACATCTAAGGGCATCACAG |
| *tnf-α*-F | ACACCTCTCAGCCACAGGAT |
| *tnf-α*-R | CCGTGTCCCACTCCATAGTT |
| *cox2*-F | CTGGAAAGGCAACACAAGC |
| *cox2*-R | CGGTGAGAGTCAGGGACAT |
| *il6*-F | CGACACACCCACTATTTACAAC |
| *il6*-R | TCCCATTTTCTGAACTGCCTCT |
| *mfn1*-F | AGAATACATGGAGGATGTGAGGA |
| *mfn1*-R | GGAGACAAAGAAAATGCGGTTAG |
| *mfn2*-F | CCTGTCGGTGGGTCTATACG |
| *mfn2*-R | ACTCCTGCTGGACTTGGTGG |
| *opa1*-F | CACAATGCCCTAGAAGACCGT |
| *opa1*-R | GATTCATCCAACTCCACCACC |
| *pgc1α*-F | GGAGTCGGAGGGTGCTTCTA |
| *pgc1α*-R | CCAAAGCCCTCACTAGCCTG |
| *nrf1*-F | ATCCGTAAGAGGCAGCAGACA |
| *nrf1*-R | AGCACCAAACACCTTGAACAC |
| *esrrα*-F | TGTTGGTGGTGTGGTAGAAAAC |
| *esrrα*-R | GGAAGCCTGGGATGTGTTT |
| *pemt-*F | ATGTACTGGGGCAGCACTG |
| *pemt-R* | AGTGAATGGTCCCTCAAATCTTA |
| *vdac1*-F | ATATGGCTTTGGGCTCATCA |
| *vdac1*-R | TGTCAGTGTTCCACTTTTCTGTG |
| For zebrafish |  |
| *18s rRNA*-F | TTCCGATAACGAACGAGAC |
| *18s rRNA*-R | GACCTGTTATTGCTCCATCT |
| *tnf-α*-F | TCTGCTTCACGCTCCATAAGACC |
| *tnf-α*-R | GCCTTGGAAGTGAAATTGCCTT |
| *cox2a*-F | ACTACCCCTGAGCTTCTCACA |
| *cox2a*-R | GATGCTGTTGATGATATCCCAGATTG |
| *il6*-F | GCGTCCTGACGTGGTATAAAG |
| *il6*-R | GTCGTTTGGTGCTGTGTTTG |
| *mfn1b*-F | GAAATCGAATCGTACACCAG |
| *mfn1b*-R | GTGCTTTTACCATTGCTTGT |
| *mfn2*-F | TCACTGCCAAGAAGAAGATC |
| *mfn2*-R | TAACAGGATCCAGCTCTTCA |
| *opa1*-F | GAAAGTTGTATTGCAGAAGG |
| *opa1*-R | CAGGTGATCTTGAGTGTTGT |
| *pgc1α*-F | CCTGAGCTTGACCTTTCTGA |
| *pgc1α*-R | GGCCTCATTTTCCTCATCTA |
| *nrf1*-F | GACGATGATGCTTATGATGA |
| *nrf1*-R | CTGTTGTCTTTTACGGATTG |
| *esrrα*-F | CTCAAAGTAGGCATGCTCAA |
| *esrrα*-R | GACACAATGATGCTGGATGA |
| *pemt-*F | ACCGGACTGTTGTGGAGGAT |
| *pemt-R* | GGCAACCACATTCCAGAAGA |

*tnfα*, tumour necrosis factor-α; *cox-2*, cyclooxygenase-2; *il6*, interleukin-6; *mfn*, mitofusion; *opa1*, optic atrophy 1; *pgc1α*, peroxisome proliferator-activated receptor gamma coactivator 1-alpha; *nrf1*, nuclear respiratory factor 1; *esrrα*, estrogen related receptor alpha; *pemt*, phosphatidylethanolamine N-methyltransferase; *vdac1*, voltage dependent anion channel 1; F, forward; R, reverse.

**Table S5 Primer sequences of plasmids construction**

| Primer | Sequences (5’-3’) |
| --- | --- |
| pcDNA3.1-PEMT-GFP-F | GGATCCACTAGTCCAGTGTGGTGGAATGCACCGAGGAGATAAGTG |
| pcDNA3.1-PEMT-GFP-R | GCCACTGTGCTGGATATCTGCAGAACTCATGCTTGCGGC |
| pcDNA3.1-PEMT-F | AACTTAAGCTTGGTACCGAGCTCGATGCACCGAGGAGATAAGTG |
| pcDNA3.1-PEMT-R | GAATTCCACCACACTGGACTAGTGTTACTCATGCTTGCGGC |
| pcDNA3.1-VDAC1-F | AACTTAAGCTTGGTACCGAGCTCGATGGCTGTACCTCCCACCTA |
| pcDNA3.1-VDAC1-R | GAATTCCACCACACTGGACTAGTGCTATGCCTGGAACTCGAG |
| pcDNA3.1-VDAC1-RFP-F | CTAGCGTTTAAACGGGCCCTCTAGAATGGCTGTACCTCCCACCTA |
| pcDNA3.1-VDAC1-RFP-R | ATATCCAGCACAGTGGCGGCCGCTGCCTGGAACTCGAG |
| pBiFC-VC155-VDAC1-F | ACCGAGATCTCTCGAGGTACCATGGCTGTACCTCCCACCTA |
| pBiFC-VC155-VDAC1-R | TTTGCACGCCGGACGGGTACCTGCCTGGAACTCGAG |
| pBiFC-VN173-PEMT-F | AAAGACGATGACGACAAGCTTATGCACCGAGGAGATAAGTG |
| pBiFC-VN173-PEMT-R | TGAATTCGCGGCCGCAAGCTTCTCATGCTTGCGGC |
| Ha-VDAC1-F | AACTTAAGCTTGGTACCGAGCTCGATGGCTGTACCTCCCACCTA |
| Ha-VDAC1-R | GAATTCCACCACACTGGACTAGTGCTAAGCGTAGTCTGGGACGTCGTATGGGTATGCCTGGAACTCGAG |
| Flag-PEMT-F | AACTTAAGCTTGGTACCGAGCTCGATGCACCGAGGAGATAAGTG |
| Flag-PEMT-R | GAATTCCACCACACTGGACTAGTGTTACTTATCGTCGTCATCCTTGTAATCCTCATGCTTGCGGC |

**Supplemental Figures**


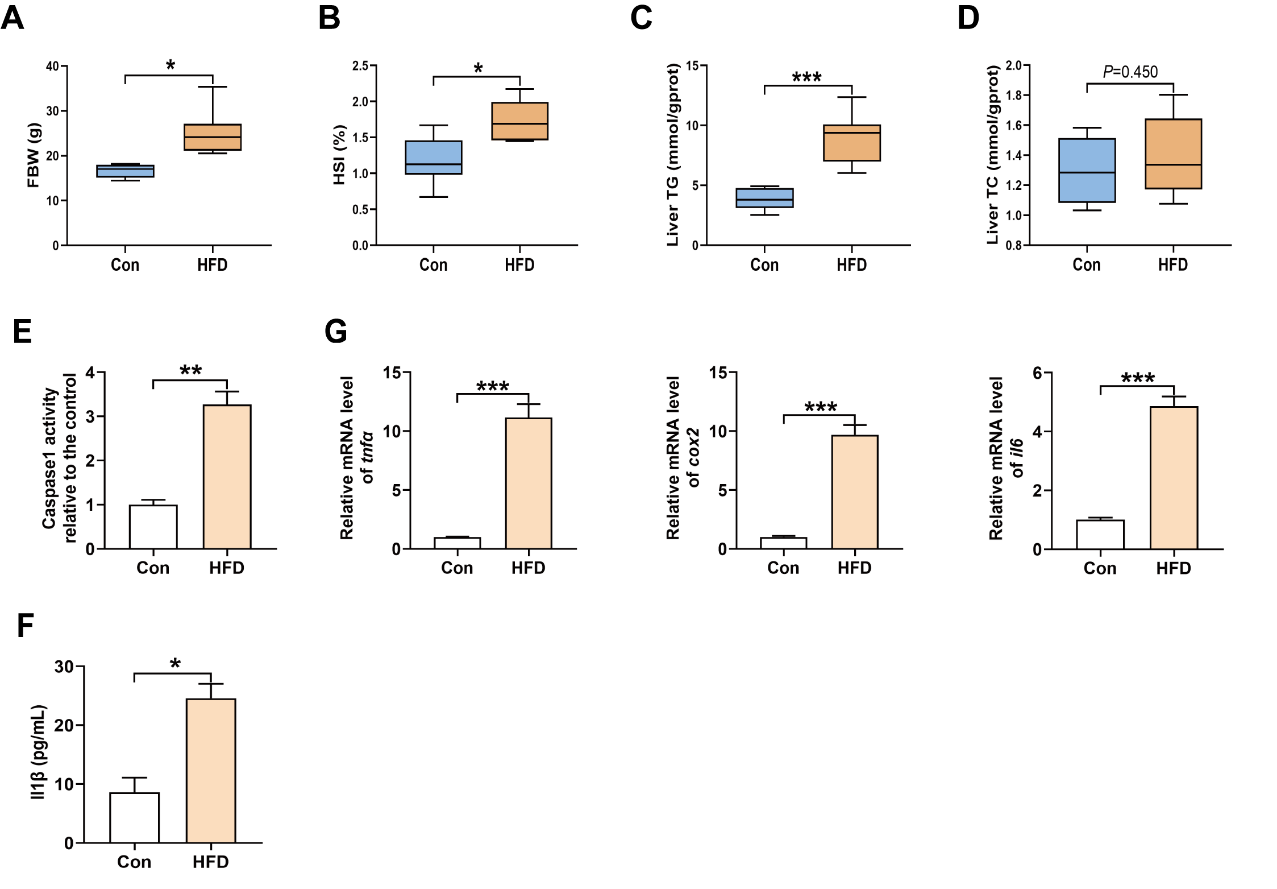


**Figure S1** Effects of high-fat diet (HFD) on (A) final body weight (*n* = 6), (B) hepatosomatic index (*n* = 6), hepatic (C) triglyceride (*n* = 6), (D) total cholesterol contents (*n* = 6), and (E) Caspase1 activity (*n* = 6), (F) serum Il1β content (*n* = 6), and (G) hepatic mRNA levels of inflammatory response (*tnfα*, *cox2*, and *il6*) of large yellow croaker (*n* = 6). The results are presented as the mean ± SEM and analyzed by independent *t*-tests. **P* < 0.05, ***P* < 0.01, and ****P* < 0.001.


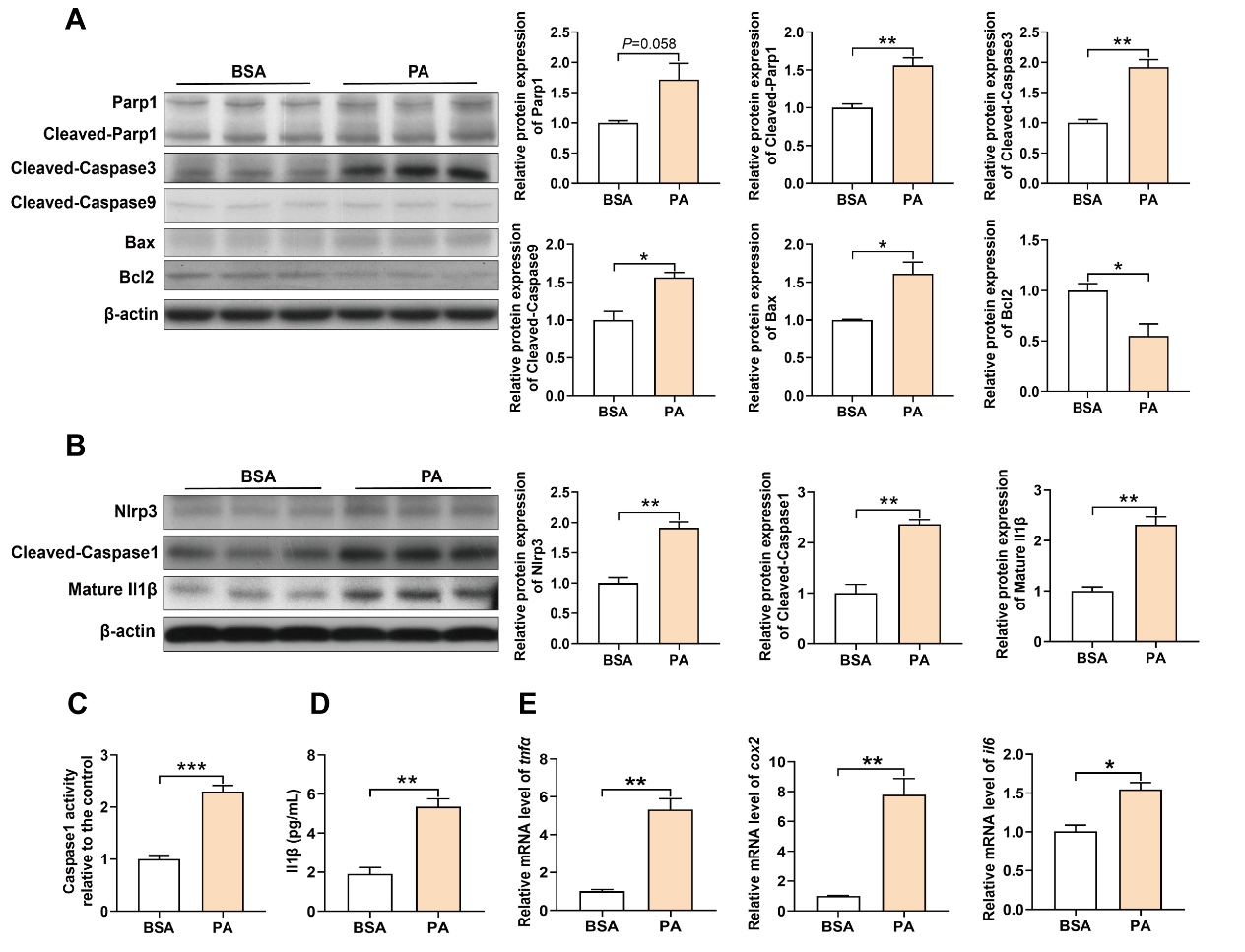


**Figure S2** Palmitic acid (PA) induces apoptosis and inflammatory responses in the hepatocytes of large yellow croaker. (A) Hepatocyte protein expression of apoptosis related protein (Parp1, Cleaved-Parp1, Cleaved-Caspase3, Cleaved-Caspase9, Bax and Bcl2) (*n* = 3), (B) hepatocyte Nlrp3 inflammasome activation related protein (Nlrp3, Cleaved-Caspase1, and mature Il1β) (*n* = 3), (C) hepatocyte Caspase1 activity, (D) culture medium Il1β content (*n* = 3), and (E) hepatocyte mRNA levels of inflammatory response (*tnfα*, *cox2*, and *il6*) after treatment with PA for 12 h (*n* = 3). The results are presented as the mean ± SEM and analyzed by independent *t*-tests. **P* < 0.05, ***P* < 0.01, and ****P* < 0.001.


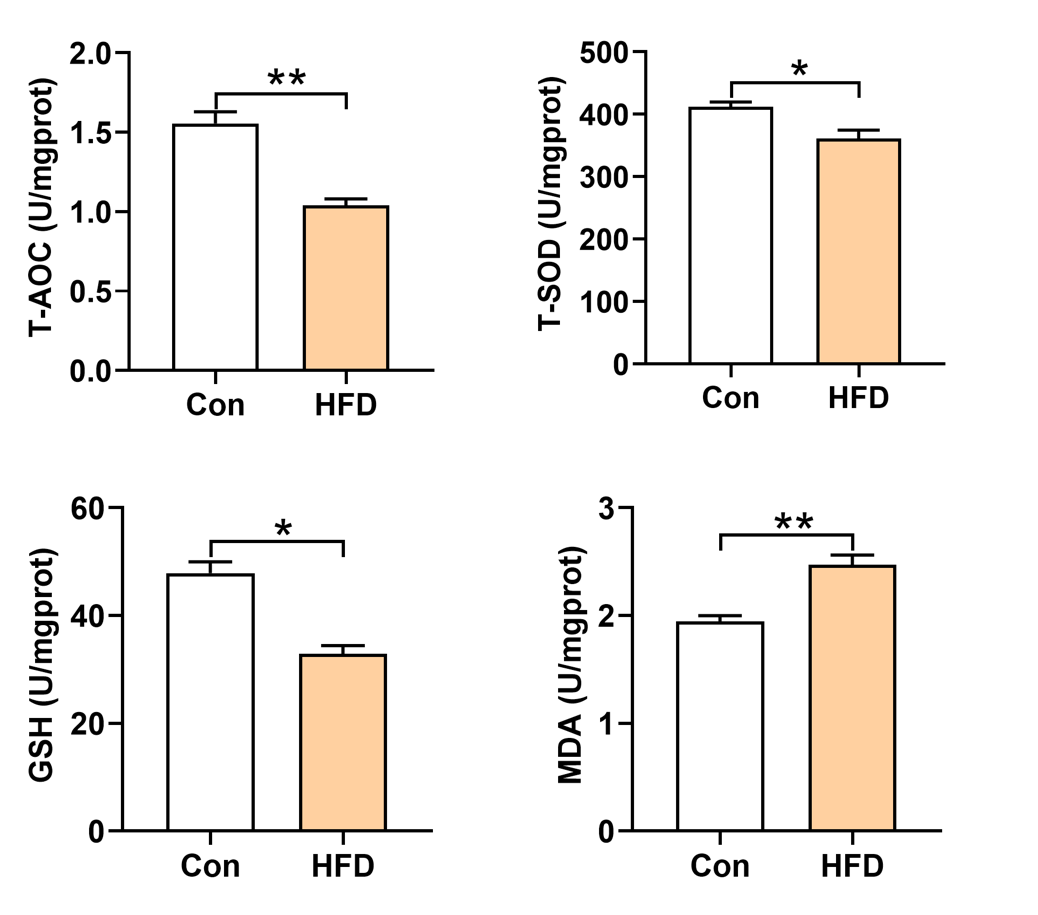


**Figure S3** Effects of HFD on antioxidant capacity in the liver of large yellow croaker (*n* = 6). T-AOC, total antioxidant capacity; T-SOD, total superoxide dismutase; GSH, glutathione; MDA, malondialdehyde. The results are presented as the mean ± SEM and analyzed by independent *t*-tests. **P* < 0.05 and ***P* < 0.01.


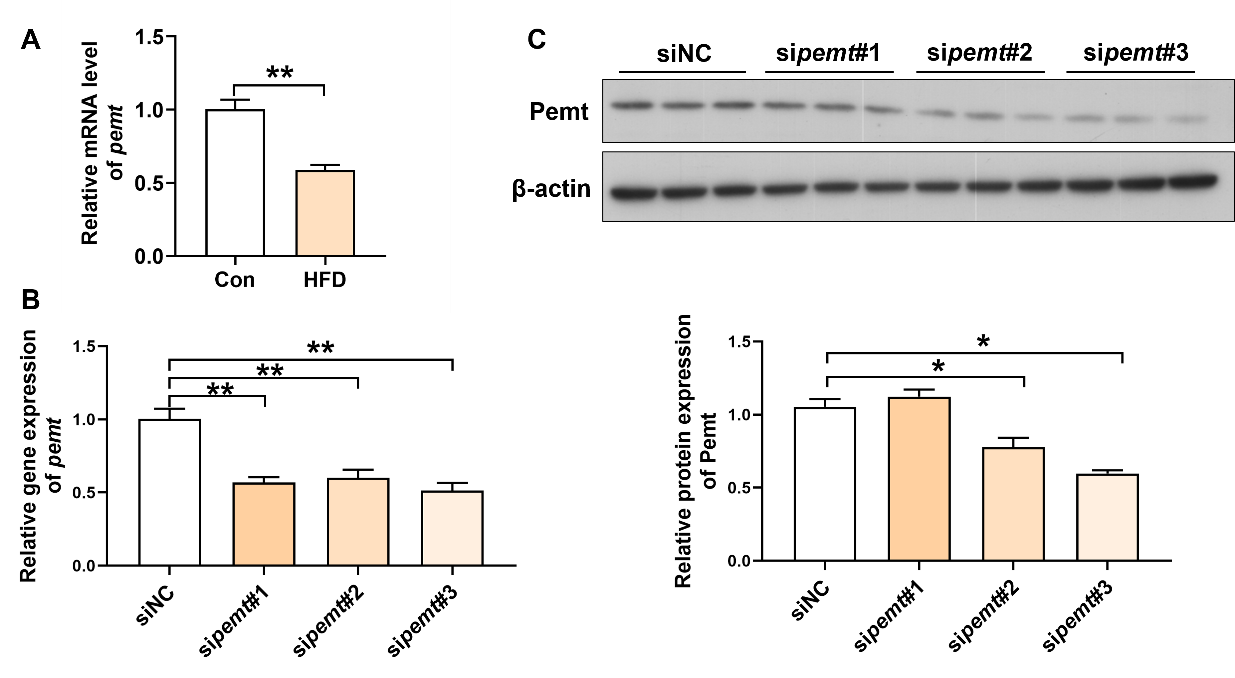


**Figure S4** Effects of HFD and *pemt* knockdown on *pemt* mRNA and Pemt protein expression in the liver and hepatocytes of large yellow croaker. (A) mRNA level of *pemt* of large yellow croaker fed HFD (*n* = 6); (B) mRNA level of *pemt* after interfered with si*pemt* for 24 h (n=3); (C) Protein expression of Pemt after interfered with si*pemt* for 48 h (n=3). The results are presented as the mean ± SEM and analyzed by independent *t*-tests. **P* < 0.05 and ***P* < 0.01.


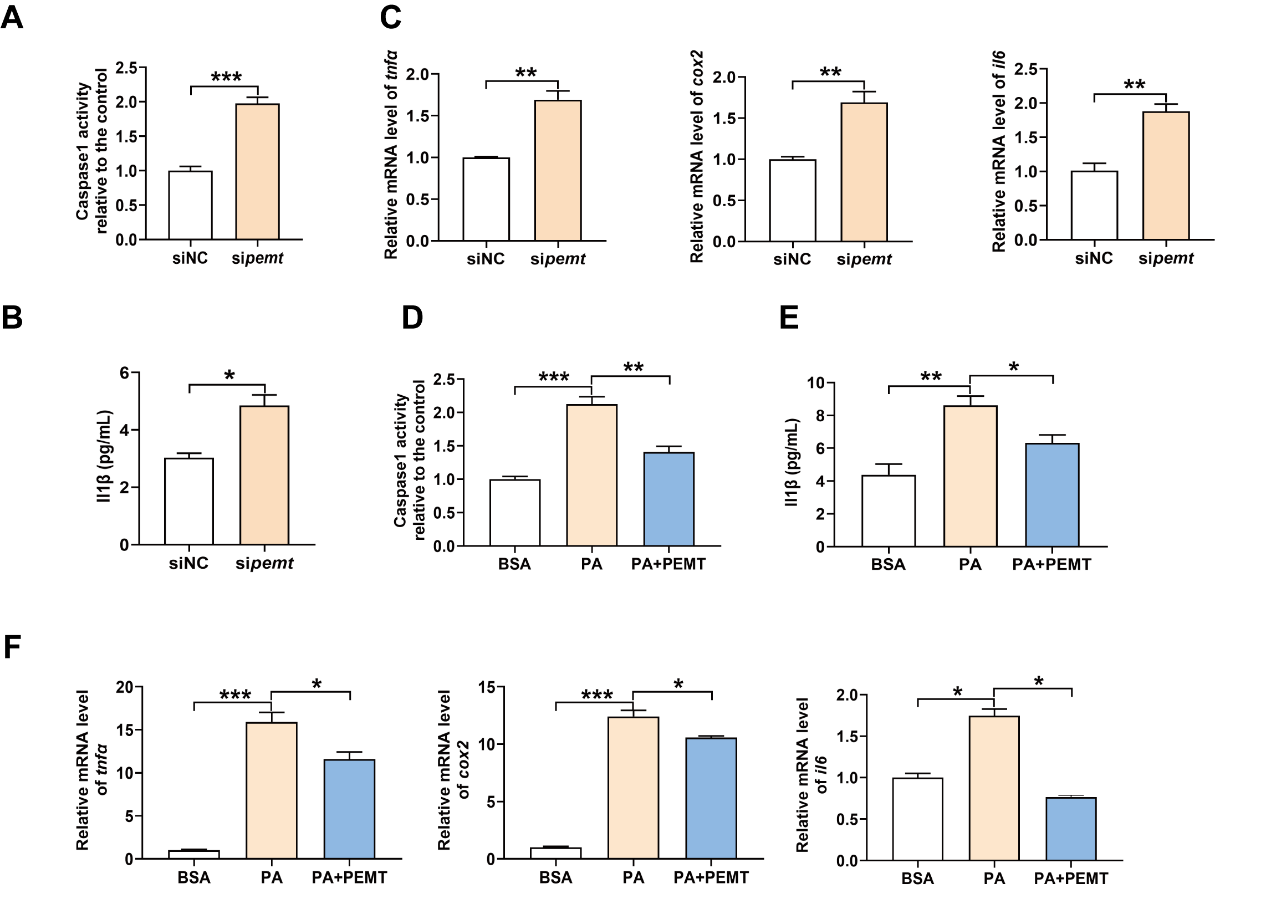


**Figure S5** Effects of (A-C) *pemt* knockdown and (D-F) PEMT overexpression on the hepatocyte Caspase1 activity, culture medium Il1β content, and hepatocyte mRNA expression of inflammatory response (*tnfα*, *cox2*, and *il6*) of large yellow croaker (*n* = 3). The results are presented as the mean ± SEM and analyzed by independent *t*-tests. **P* < 0.05, ***P* < 0.01, and *** *P* < 0.001.


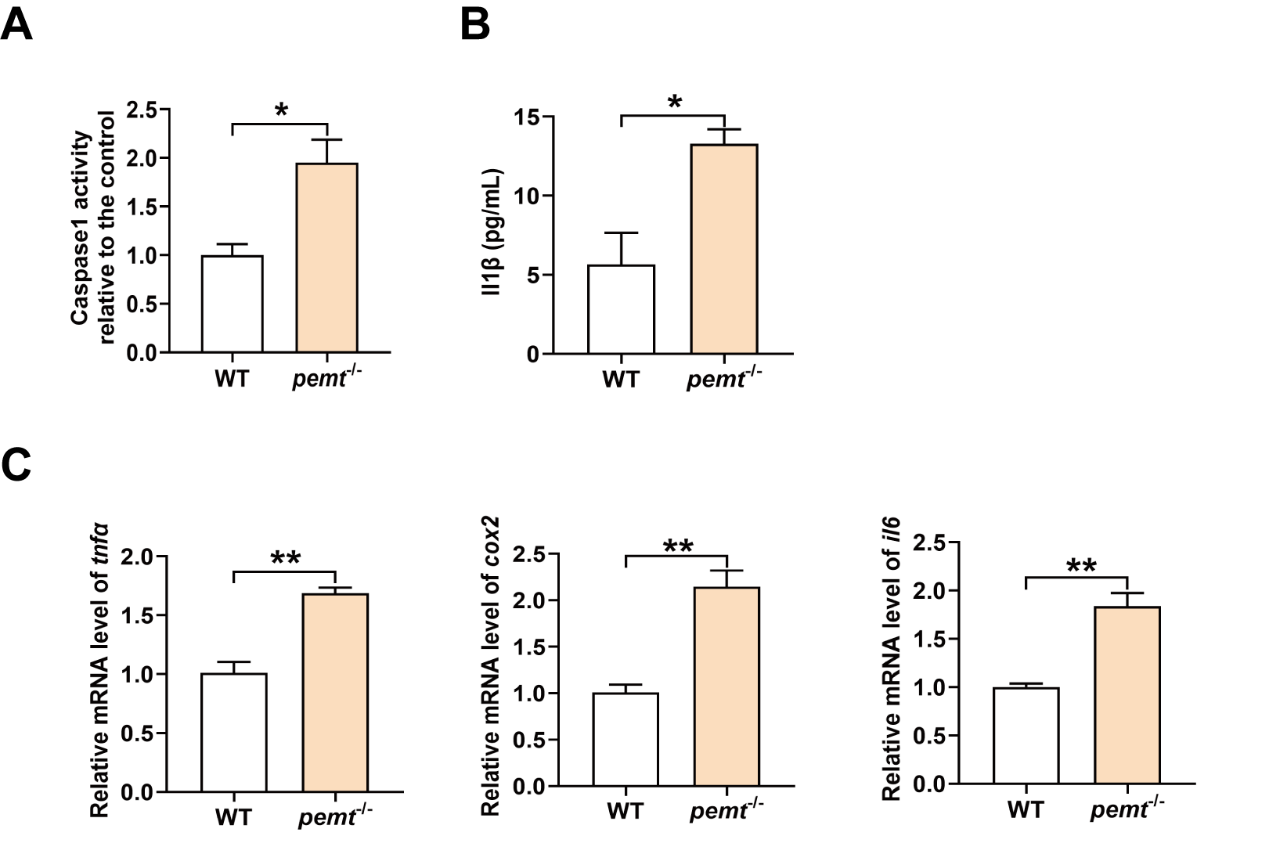


**Figure S6** Effects of *pemt* knockout on the (A) hepatic Caspase1 activity, (B) serum Il1β content, and (C) hepatic mRNA expression of inflammatory response (*tnfα*, *cox2*, and *il6*) of zebrafish (*n* = 6). The results are presented as the mean ± SEM and analyzed by independent *t*-tests. **P* < 0.05 and ***P* < 0.01.


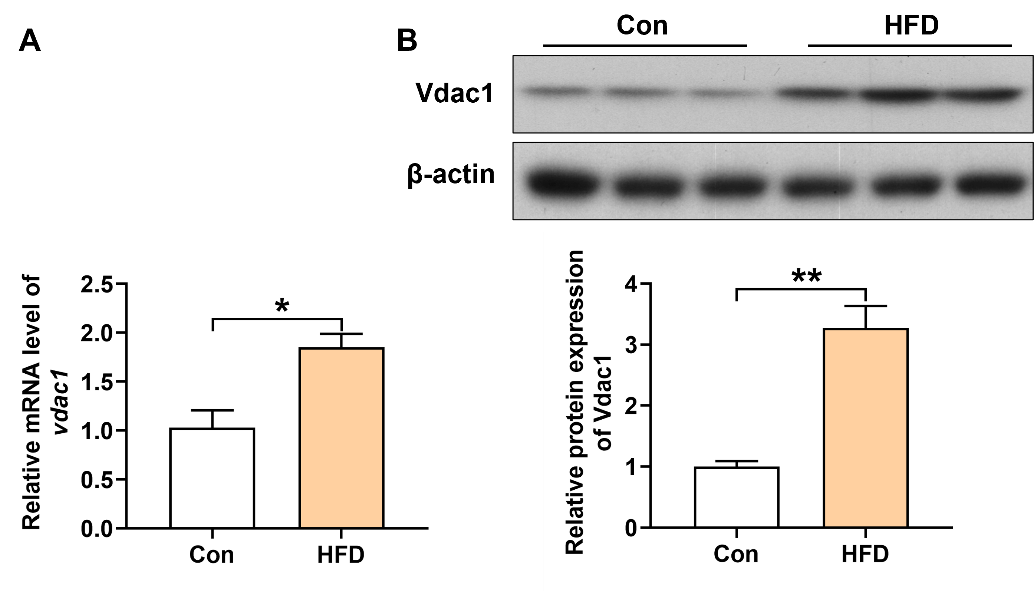


**Figure S7** HFD increases (A) *vdac1* mRNA and (B) Vdac1 protein expression in the liver of large yellow croaker. The results are presented as the mean ± SEM and analyzed by independent *t*-tests (*n* = 6). **P* < 0.05 and ***P* < 0.01.


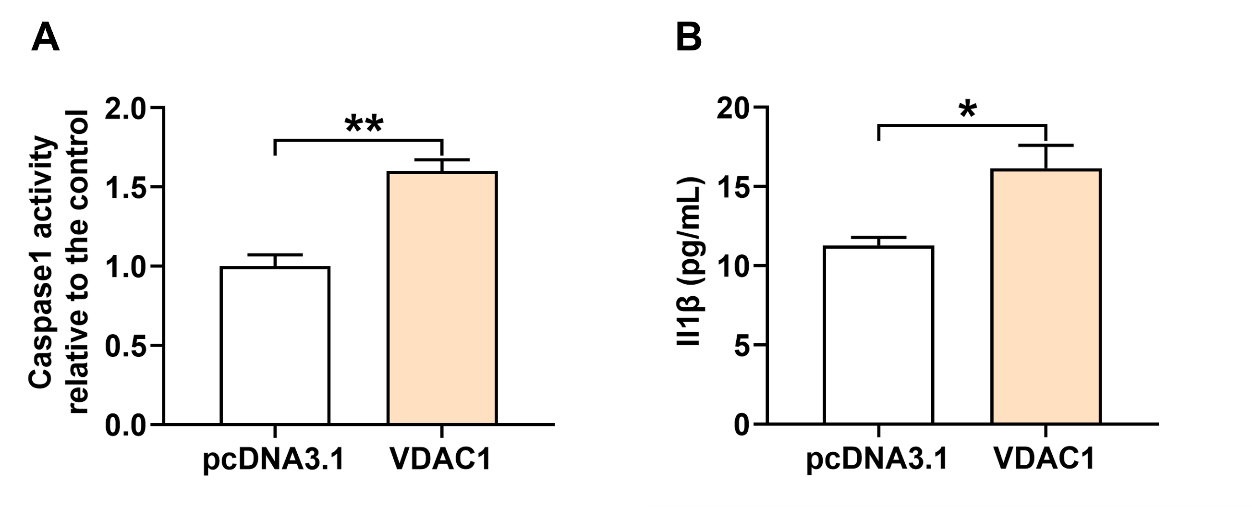


**Figure S8** Effects of VDAC1 overexpression on the (A) hepatocyte Caspase1 activity and (B) culture medium Il1β content of large yellow croaker. The results are presented as the mean ± SEM and analyzed by independent *t*-tests (*n* = 3). **P* < 0.05 and ***P* < 0.01.


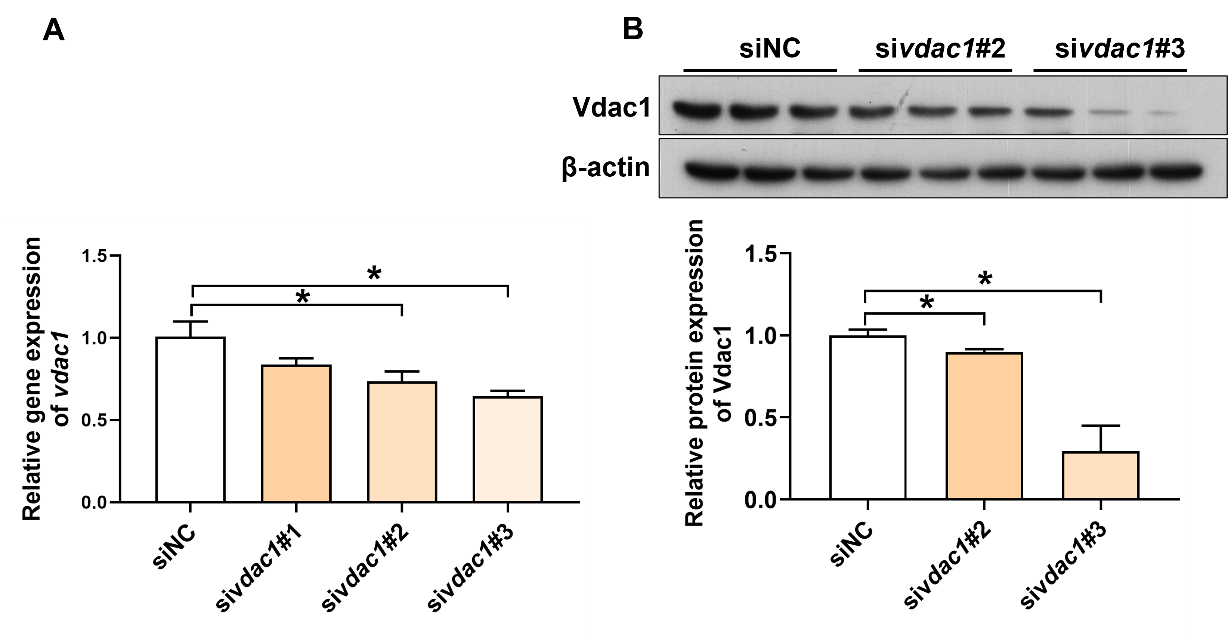
**Figure S9** Effects of knockdown of *vdac1* on the *vdac1* mRNA and Vdac1 protein expression in the hepatocytes of large yellow croaker. (A) mRNA expression of *vdac1* after interfered with si*vdac1* for 24 h (*n* = 3). (B) Protein expression of Vdac1 after interfered with si*vdac1* for 48 h (*n* = 3). The results are presented as the mean ± SEM and analyzed by independent *t*-tests. **P* < 0.05.

**
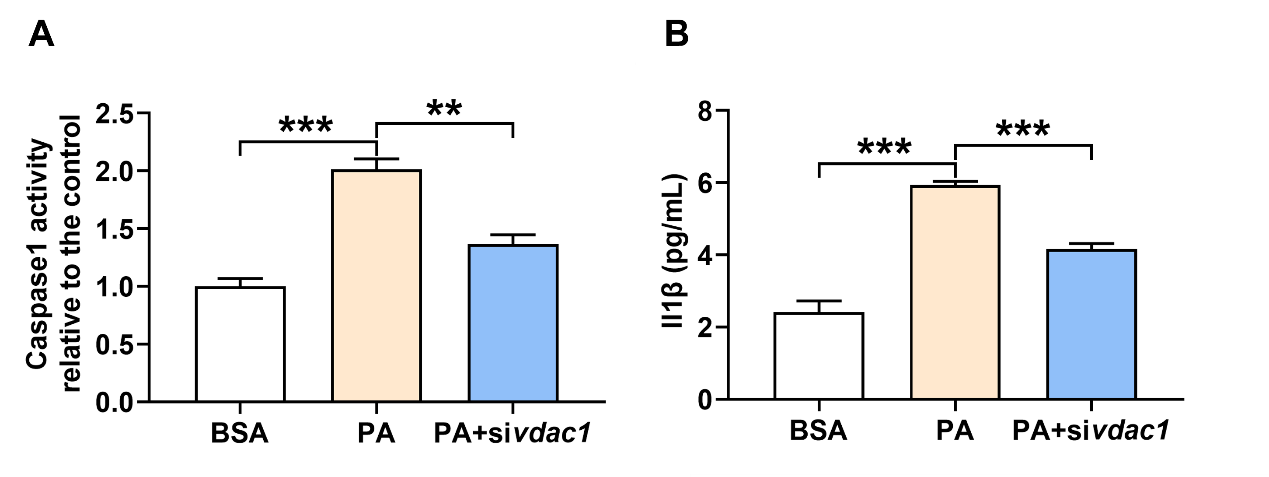
**

**Figure S10** Knockdown of *vdac1* attenuated PA-induced increase in hepatocyte Caspase1 activity and culture medium Il1β content of large yellow croaker. The results are presented as the mean ± SEM and analyzed by independent *t*-tests (*n* = 3). ** *P* < 0.01 and *** *P* < 0.001.

**
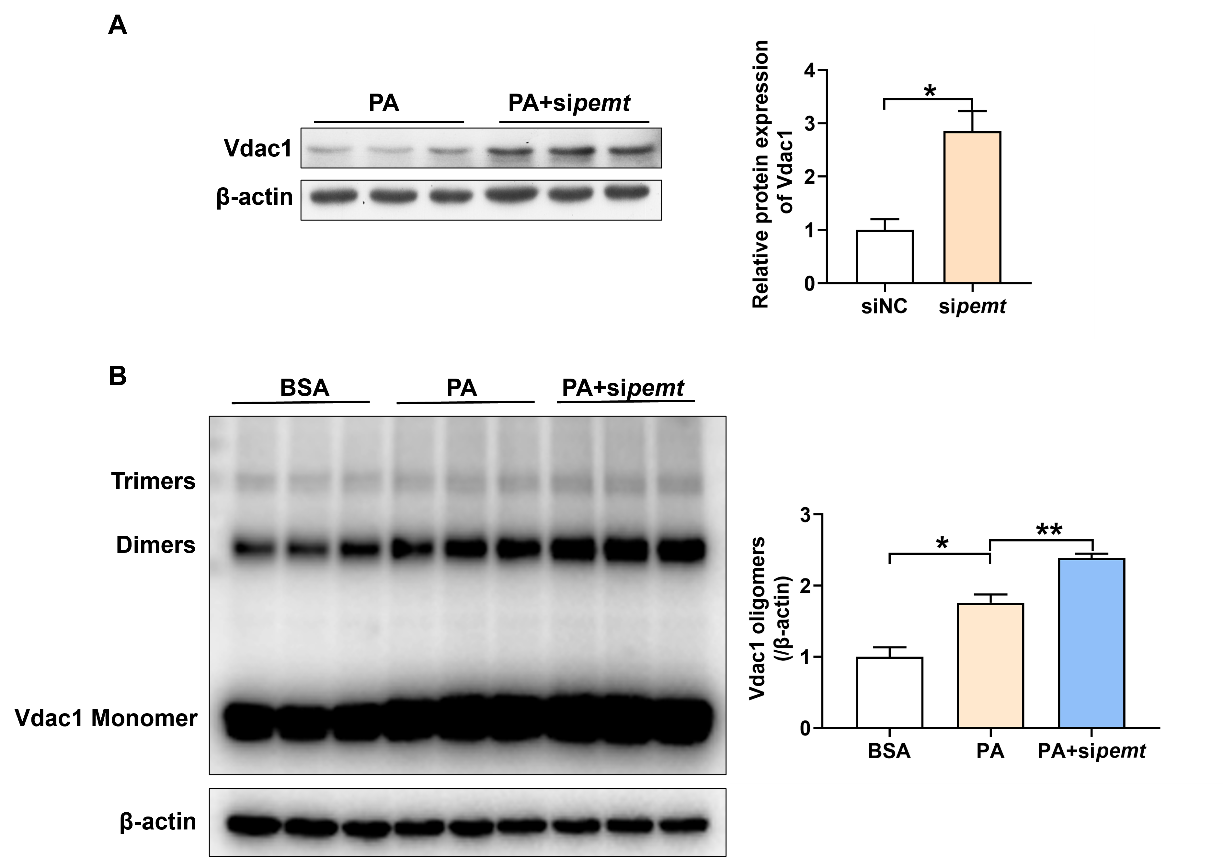
**

**Figure S11** Knockdown of *pemt* aggravated PA-induced increase in protein expression and oligomerization of Vdac1 in the hepatocytes of large yellow croaker. (A) Protein expression of Vdac1 after interfered with si*pemt* for 36 h followed by PA treatment for 12 h (*n* = 3). (B) Oligomerization of Vdac1 after interfered with si*pemt* for 36 h followed by PA treatment for 12 h (n = 3). The results are presented as the mean ± SEM and analyzed by independent *t*-tests. * *P* < 0.05 and ** *P* < 0.01.


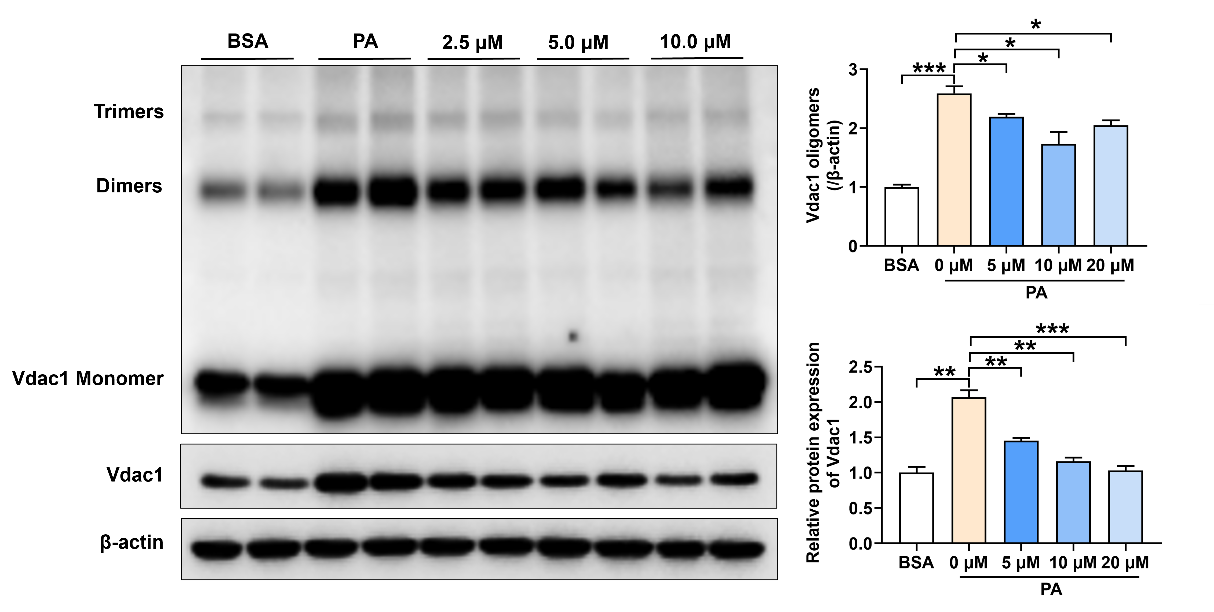


**Figure S12** VBIT-12 attenuated the PA-induced increase in both oligomerization and protein expression of Vdac1 in large yellow croaker hepatocytes. The results are presented as the mean ± SEM and analyzed by independent *t*-tests (*n* = 3). * *P* < 0.05, ** *P* < 0.01, and *** *P* < 0.001.

**
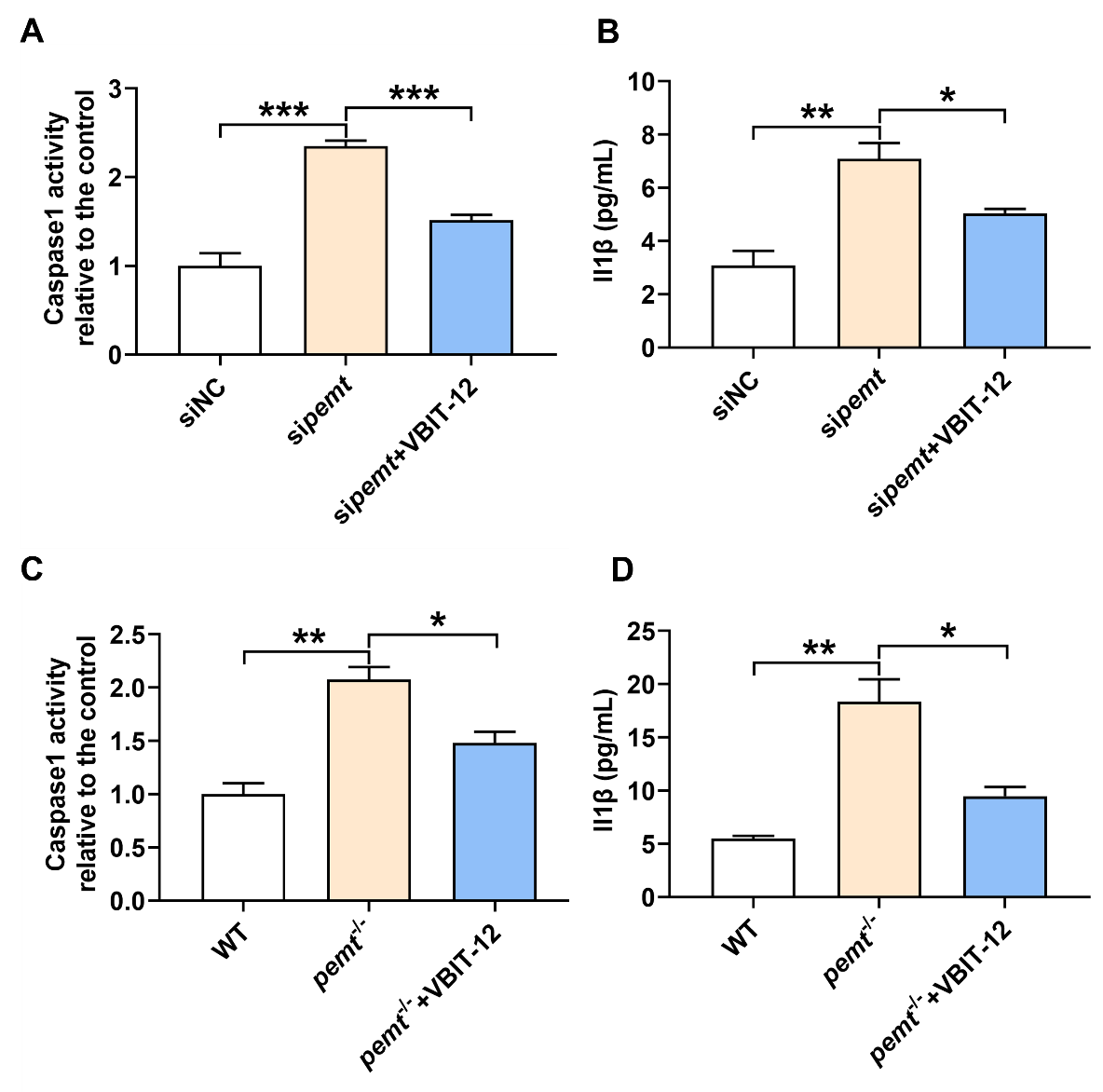
**

**Figure S13** VBIT-12 attenuated *pemt* knockdown- and knockout-induced increases in Caspase-1 activity and Il1β secretion in large yellow croaker hepatocytes and *pemt* knockout zebrafish. (A) Caspase1 activity in large yellow croaker hepatocytes (*n* = 3); (B) Il1β content in culture medium of large yellow croaker hepatocytes (*n* = 3); (C) Caspase1 activity in the liver of *pemt* knockout zebrafish (*n* = 3); and (D) Il1β content in the serum of *pemt* knockout zebrafish (*n* = 3). The results are presented as the mean ± SEM and analyzed by independent *t*-tests. * *P* < 0.05, ** *P* < 0.01, and *** *P* < 0.001.
